# Structures of heat shock factor trimers bound to DNA

**DOI:** 10.1016/j.isci.2021.102951

**Published:** 2021-08-05

**Authors:** Na Feng, Han Feng, Sheng Wang, Avinash S. Punekar, Rudolf Ladenstein, Da-Cheng Wang, Qinghua Zhang, Jingjin Ding, Wei Liu

**Affiliations:** 1 National Laboratory of Biomacromolecules, Institute of Biophysics, Chinese Academy of Sciences, Beijing 100101, China; 2 University of Chinese Academy of Sciences, Beijing 100049, China; 3 CAS Key Laboratory of Infection and Immunity, Institute of Biophysics, Chinese Academy of Sciences, Beijing 100101, China; 4 College of Life Science and Technology, Huazhong University of Science and Technology, Wuhan, Hubei 430074, China; 5 Department of Biosciences and Nutrition, Karolinska Institutet NEO, 14183 Huddinge, Sweden; 6 Department of Obstetrics and Gynecology, Daping Hospital, Army Medical University of PLA, Chongqing 400042, China; 7 Institute of Immunology, Army Medical University, Chongqing 400038, China

**Keywords:** Molecular Structure, Structural biology, Protein structure aspects

## Abstract

Heat shock factor 1 (HSF1) and 2 (HSF2) play distinct but overlapping regulatory roles in maintaining cellular proteostasis or mediating cell differentiation and development. Upon activation, both HSFs trimerize and bind to heat shock elements (HSEs) present in the promoter region of target genes. Despite structural insights gained from recent studies, structures reflecting the physiological architecture of this transcriptional machinery remains to be determined. Here, we present co-crystal structures of human HSF1 and HSF2 trimers bound to DNA, which reveal a triangular arrangement of the three DNA-binding domains (DBDs) with protein-protein interactions largely mediated by the wing domain. Two structural properties, different flexibility of the wing domain and local DNA conformational changes induced by HSF binding, seem likely to contribute to the subtle differential specificity between HSF1 and HSF2. Besides, two more structures showing DBDs bound to “two-site” head-to-head HSEs were determined as additions to the published tail-to-tail dimer-binding structures.

## Highlights

• Three structures of HSF-DBD bound to DNA comprising three HSE repeats were determined
• Two structures of HSF-DBD bound to two-site head-to-head HSEs were determined
• Structural determinants for auxiliary HSF-HSE interactions were discovered
• A DNA-shape readout mechanism exploited by the HSF family members was revealed

## Graphical abstract

**Figure:**
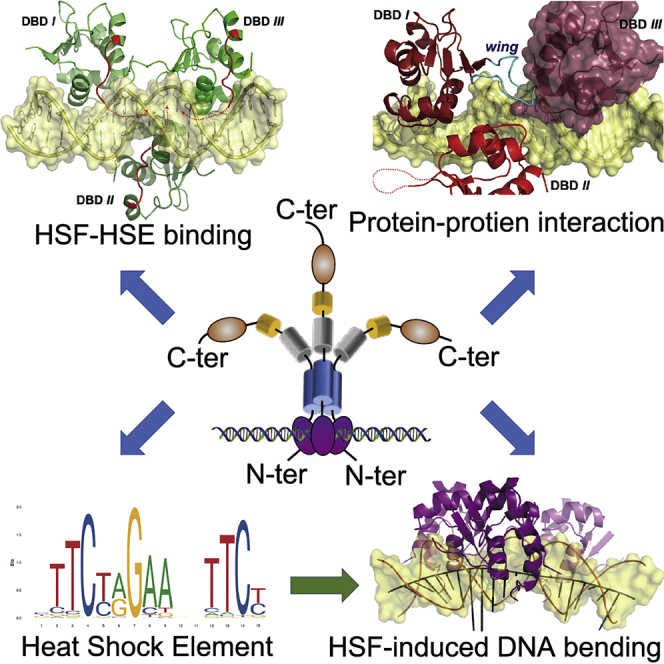

## Introduction

All living cells and tissues are constantly challenged by acute or chronic stress. Exposure to various protein-damaging stimuli, such as elevated temperature, heavy metals, toxins and hypoxia, and various pathologic conditions, such as cancer, ischemia, infections, and inflammation, induces cellular responses for protein homeostasis (proteostasis) maintenance. One of the major cytoprotective responses is the evolutionarily conserved heat shock response (HSR) characterized by upshifted expression level of heat shock proteins (HSPs). Most HSPs function as molecular chaperones that are essential for prevention of protein aggregation and directing ubiquitin-proteasomal degradation (Vihervaara and Sistonen, 2014; Westerheide et al., 2012). Disruption of proteostasis by blocking the synthetic pathway of HSPs results in accumulation of misfolded and aggregated protein species in cells, and inclusions indicative of neurodegenerative disease (Gidalevitz et al., 2010; Neef et al., 2011), whereas constitutively elevated levels of HSPs is considered as a mechanism underlying deregulated cell proliferation and suppressed cell death, which are characteristics for tumorgenesis (Dai et al., 2007; Scherz-Shouval et al., 2014).

In eukaryotes, HSP expression is regulated at the transcriptional level through *cis*-acting nucleotide sequences called heat shock elements (HSEs), which are multiply present in the promoter region of heat shock genes (Akerfelt et al., 2010a; Bjork and Sistonen, 2010). Typical HSEs are composed of at least three alternating, inverted repeats of the pentameric sequence 5′-nGAAn-3′ (n denotes any nucleotide), where guanine is the most conserved nucleotide (Amin et al., 1988). For every pair of inverted repeats, there are two possible sequence arrangements, referred to as the head-to-head (nGAAnnTTCn) and the tail-to-tail orientation (nTTCnnGAAn) (Perisic et al., 1989). The TTC triplet and downstream GAA are often separated by a pyrimidine-purine dinucleotide, but the nucleotides preceding downstream TTC in a head-to-head arrangement are unrestrained (Figure S1A) (Akerfelt et al., 2010a). Despite all these well-characterized consensus elements, recent comprehensive ChIP-seq experiments have demonstrated that HSEs are highly diverse with variable primary sequences, lengths, and orientations of the nGAAn repeats in sequenced genomes (Guertin and Lis, 2010; Vihervaara et al., 2013).

Upon transactivation, HSEs are recognized and bound by *trans-*acting factors called heat shock factors (HSFs). Although HSFs were originally identified as transcriptional regulators of the HSR, their targets are not confined to HSP-encoding genes since HSEs are present in a plethora of genes constitutively transcribed in various cellular contexts (Guisbert et al., 2013; Riva et al., 2012). The human genome encodes six HSF proteins, HSF1, HSF2, HSF4, HSF5, HSFX, and HSFY, differing from each other in chromosomal loci, expression profiles, and physiological functions (Gomez-Pastor et al., 2018). HSF1 is the major regulator of the HSR, whereas HSF2 is more associated with development and cell differentiation (Bjork and Sistonen, 2010; Pirkkala et al., 2001); both proteins are present in most tissues and cell types. HSF1 exists as a monomer under normal conditions, whereas inactive HSF2 predominantly remains in a dimeric state (Bjork and Sistonen, 2010; Pirkkala et al., 2001). When activated, both HSFs trimerize and are translocated from cytosol to the nucleus (Akerfelt et al., 2010b; Bjork and Sistonen, 2010; Pirkkala et al., 2001; Westerheide et al., 2012), probably under regulation of a number of post-translational modifications such as phosphorylation, acetylation, and SUMOylation (Anckar et al., 2006; Kline and Morimoto, 1997; Westerheide et al., 2009). Despite their distinct functions, recent studies have demonstrated that these two factors possess overlapping functions and may cooperatively regulate HSR through interplay or direct interactions between them (Ostling et al., 2007; Sandqvist et al., 2009).

HSFs are modular proteins composed of a DNA-binding domain (DBD), a coiled-coil trimerization domain, a regulatory domain, and a transactivation domain from the N- to C-terminal end (Pirkkala et al., 2001; Vihervaara and Sistonen, 2014; Westerheide et al., 2012). HSFs recognize and bind HSEs through the DBD, which is the most conserved and only structurally characterized domain (Figure S1B). The adjacent oligomerization domain, also referred to as HR-A/B, is characterized by the presence of hydrophobic heptad repeats in the amino acid sequence and mediates trimerization through the formation of a three-bundled coiled coil among protein monomers (Wu, 1995). HSF trimerization produces a synergistic effect among DBDs that significantly increases the affinity for HSE binding (Sorger and Nelson, 1989). One nGAAn repeat is contacted by an individual DBD, and thus an HSF trimer optimally binds three repeats in an HSE.

Crystal and solution structures of HSF-DBD from *Kluyveromyces lactis*, *Drosophila melanogaster*, and human displayed a winged helix-turn-helix fold in this domain (Feng et al., 2016b; Littlefield and Nelson, 1999; Vuister et al., 1994; Xiao et al., 2020). More mechanistic insights into HSF-HSE recognition, however, were obtained from recently reported co-crystal structures of DBD in human HSF1 and HSF2 bound to tail-to-tail pentameric repeats and head-to-tail satellite III (SatIII) repeats (Jaeger et al., 2016; Neudegger et al., 2016). Upon DNA binding, the second helix in the helix-turn-helix motif (α3, often referred to as “the recognition helix”) inserts into the DNA major groove and contributes predominant interactions with the nGAAn consensus motif. The sequence specificity of protein-DNA recognition is largely mediated by a pair of bidentate hydrogen bonds formed between a highly conserved arginine and the guanine in a GAA triplet. Dissimilar to most other DNA-binding proteins containing a winged helix-turn-helix motif, the wing in HSFs does not contact the DNA. Instead, it is proposed to mediate protein-protein interactions probably among DBDs within an HSF trimer or between neighboring trimers (Jaeger et al., 2016; Littlefield and Nelson, 1999; Neudegger et al., 2016), which, however, needs to be supported by more evidence. Although the coiled coil following DBD is absent in the crystal structures, it is proposed to be positioned at the opposite side of the DNA double helix, thus allowing HSF to embrace DNA (Jaeger et al., 2016; Neudegger et al., 2016).

Despite the acquired mechanistic insights, the architecture of the HSF-HSE complex assembled in physiological context is not disclosed because only HSF dimers were presented in the published structures. Co-crystal structures revealing an HSF trimer bound to an intact HSE containing three nGAAn repeats are indispensable for understanding how the cooperative binding among the three DBDs is fulfilled. Jaeger *et al.* have attempted to crystallize HSF2-DBD with a three-site HSE but failed to obtain a trimer-binding structure (Jaeger et al., 2016). In this study, we successfully determined three co-crystal structures of three DBDs in human HSF1 and HSF2 occupying three binding sites in an HSE after systematic optimizations with regard to DNA lengths and terminal compositions. Besides, two complex structures showing DBDs bound to head-to-head repeats were solved as additions to the reported dimer-binding structures.

## Results

### Structure of the DBD bound to “two-site” head-to-head HSEs

Previous biochemical studies have shown that HSF1 and HSF2 bind to a two-site tail-to-tail HSE with higher affinity than to a head-to-head counterpart (Tateishi et al., 2009), but the structural basis for this difference is not well understood owing to the lack of head-to-head structures. To address this question, we first determined two structures: HSF2-DBD (residues 7–112) bound to a 12-bp oligonucleotide and HSF1-DBD (residues 15–120) bound to a 14-bp oligonucleotide (Table S1) at resolutions 1.7 and 2.0 Å (Table 1). The DNA molecules present in both crystals comprised blunt-ended head-to-head sequences but packed with different orientations in the crystal lattice: head-to-tail versus head-to-head in the HSF2 and HSF1 structures, respectively (Figure S2). All base pairs were well defined in electron density except for an invisible terminal pair present in the HSF1 structure.

**Figure 1:**
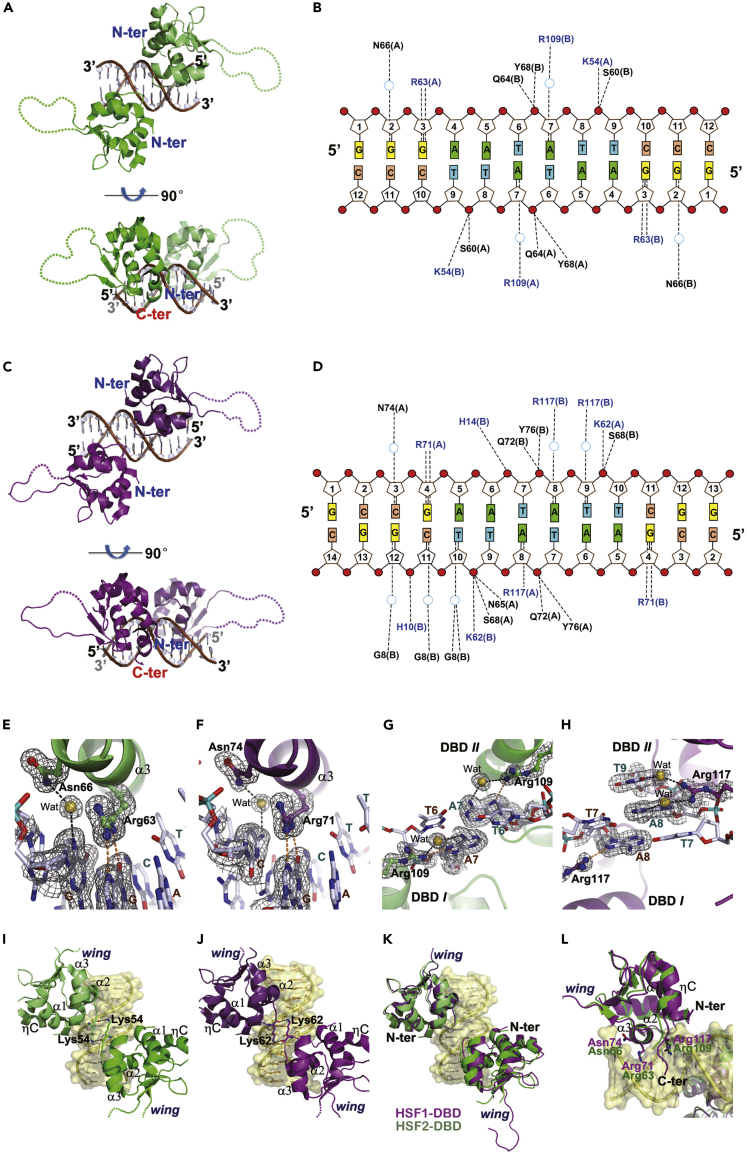
Interactions of the DBDs in human HSF1/2 with two-site HSEs in the head-to-head orientation (A–D) The crystal structures of HSF2-DBD bound to 12-bp (A) and HSF1-DBD bound to 14-bp cognate DNA (C), and corresponding schematic representations of protein-DNA interactions (B and D). (E and F) The bidentate hydrogen bond of a highly conserved Arg residue with the guanine in GAA, and a solvent-mediated hydrogen bond between an Asn residue and the nucleobase preceding GAA in either HSF2 (E) or HSF1 (F) structure. (G and H) Other hydrogen bonds formed along the major groove between a conserved C-terminal Arg residue with nucleobases between two GAA triplets in either HSF2 (G) or HSF1 (H) structure. (I and J) The dimer interface between the two DBDs in either HSF2 (I) or HSF1 (J) structure. (K) Superimposition of HSF1 and HSF2 bound to cognate DNA (L) Close view of overlaid DBDs. In all panels, HSF1 and HSF2 are colored in purple and green, respectively. The direct and solvent-mediated hydrogen bonds are represented by orange and black dashed lines, respectively. The 2*F*_o_ – *F*_c_ electron density contoured at 1.0 σ is shown as gray mesh.

Both structures showed two DBD copies occupying two binding sites with a dimer interface formed over the minor groove (Figures 1A and 1C). Similar to the reported structures containing tail-to-tail HSEs, all sequence-specific interactions occur in the major groove and are principally donated by the bidentate hydrogen bonds between an invariant Arg residue (Arg63 in HSF2 or Arg71 in HSF1) and the guanine in the GAA triplet (Figures 1B and 1D). Other two solvent-mediated hydrogen bonds, one bridging a neighboring conserved asparagine (N66 in HSF2 or N74 in HSF1) and the nucleobase preceding GAA (Figures 1E and 1F) and the other linking a C-terminal arginine (Arg109 in HSF2 or Arg117 in HSF1) and the nucleobase preceding TTC in the complementary strand (Figures 1G and 1H), make auxiliary contributions to the sequence-specific contacts. All these hydrogen bonds are conserved in the structures containing two-site HSEs in either head-to-head or tail-to-tail arrangement (Jaeger et al., 2016; Neudegger et al., 2016), thus establishing a sequence-readout mechanism.

Despite the convergent binding manners, significant differences in protein-protein interactions were observed when HSFs binds to the two differently oriented HSEs. The adjacent DBDs in our structures are related by fewer contacts, generating a smaller buried interface area than those bound to tail-to-tail HSEs, e.g., 85 Å^2^ versus 144 Å^2^ in the HSF2 structures. The dimerization is weakly maintained by contacts between the short turns present in the helix-turn-helix motif, where only one residue (Lys54 in HSF2 and Lys62 in HSF1) is involved in forming a 2-fold symmetric pair of hydrogen bonds (Figures 1I and 1J). The looser dimer contacts observed in these structures coincide with the distance between the two guanines in GAA, which are separated by six nucleotides in the head-to-head motif versus only two in the tail-to-tail motif. Notably, the wing domain in both DBDs remains unstructured in our structures, but obviously stretched away from the dimer interface to opposite directions (Figures 1I and 1J). Apparently, they are not involved in dimer contacts, dissimilar to those in the HSF-DBD dimer bound to tail-to-tail HSEs (Jaeger et al., 2016; Neudegger et al., 2016).

Although the DBD monomers remain in strict structural convergence (r.m.s.d. of 0.23–0.45 Å on C_α_ atoms), subtle but significant deviations exist in dimer structures between HSF1 and HSF2. Upon superimposition of the DNA backbones, one pair of DBD counterparts was well overlaid but the other was imperfect. In comparison with HSF2, one HSF1 monomer slightly moved away along the DNA axis (Figure 1K). The relative movement reflects a more extended binding manner of the HSF1 dimer, or in other words, HSF1 occupying a longer DNA segment than HSF2. This divergence may account for the different crystallization behaviors observed in our trials: HSF2 crystallized together with a 12-bp oligonucleotide but HSF1 did not unless with a longer DNA duplex. Despite the overall disagreement, the recognition helix in both HSFs is coherently positioned and orientated. The conserved Arg residue close to the C terminus, albeit imperfectly overlaid, consistently remains involved in protein-DNA interactions (Figure 1L).

### Structure of HSF2 trimer bound to a “three-site” HSE

HSF2-DBD was crystallized in complex with a canonical three-site HSE, 21 bp with a 5′-overhang (Table S1). The structure refined at 1.75 Å (Table 1) reveals an HSF2 trimer bound to the three nGAAn repeats in unambiguous electron density. The DNA in the crystal was packed in a head-to-tail manner, where the 5′-overhung adenine in one strand formed a standard Watson-Crick base pair with the 5′-overhung thymine in the complementary strand from a neighboring asymmetric unit (Figure S3). Such a packing mode leaves enough room to properly accommodate three protein monomers.

**Table 1:** Data collection and refinement statistics of HSF1/2-DBD in complex with DNA duplex

	HSF2-HtH HSE	HSF1-HtH HSE	HSF2-3site HSE	HSF1-3site HSE #1	HSF1-3site HSE #2
**DNA composition**	12 bp (blunt end)	14 bp (blunt end)	21 bp (sticky end)	23 bp (sticky end)	24 bp (sticky end)

**Data collection**					

Space group	*C*2	*P*2_1_2_1_2_1_	*P*22_1_2_1_	*P*2_1_2_1_2_1_	*P*2_1_2_1_2_1_
Cell dimensions					
*a*, *b*, *c* (Å)	92.27, 38.47, 38.65	42.22, 62.77, 97.35	46.64, 67.06, 139.65	86.72, 93.72, 142.37	85.56, 94.71, 142.26
*α, β, γ* (°)	90, 100.740, 90	90, 90, 90	90, 90, 90	90, 90, 90	90, 90, 90
Resolution (Å)	45.34-1.7 (1.76 - 1.7)	50.0-2.0 (2.03 - 2.0)	69.82 -1.75 (1.84 - 1.75)	86.72 - 2.4 (2.53 - 2.4)	85.56 - 2.36 (2.49 - 2.36)
*R*_pim_	0.034 (0.150)	0.054 (0.232)	0.033 (0.401)	0.030 (0.317)	0.028 (0.383)
*I* / *σ(I)*	25.4 (6.4)	15.5 (3.0)	13.5 (2.1)	14.5 (2.2)	14.5 (2.0)
Completeness (%)	94.0 (67.9)	99.8 (99.9)	98.0 (87.6)	99.7 (99.3)	99.9 (100.0)
Redundancy	7.44 (7.44)	6.0 (5.3)	10.4 (5.4)	12.1 (11.0)	13.1 (13.8)

**Refinement**					

Resolution (Å)	37.98-1.7	35.03-2.0	69.82 – 1.75	71.19 −2.4	63.49 -2.36
No. reflections	13957	17803	44148	41724	43397
*R*_work_ / *R*_free_	0.179/0.209	0.183/0.222	0.172/0.205	0.195/0.225	0.186/0.224
No. atoms					
Protein	787	1685	2499	4820	4808
DNA	243	530	855	1874	1956
Solvent/Ions	172	309	441	396	162
*B*-factors					
Protein	26.84	26.08	30.43	49.88	60.18
DNA	21.35	31.47	33.22	58.53	72.41
Solvent/ion	37.76	35.74	38.03	45.67	47.76
R.m.s. deviations					
Bond lengths (Å)	0.005	0.006	0.007	0.006	0.007
Bond angles (°)	0.740	0.874	0.969	0.971	1.108
Ramachandran plot					
Favored (%)	96.63	95.41	97.53	96.44	96.06
Allowed (%)	3.37	4.59	2.47	3.38	3.94
Disallowed (%)	0	0	0	0.18	0

*Values in parentheses are for highest-resolution shell.

**Figure 2:**
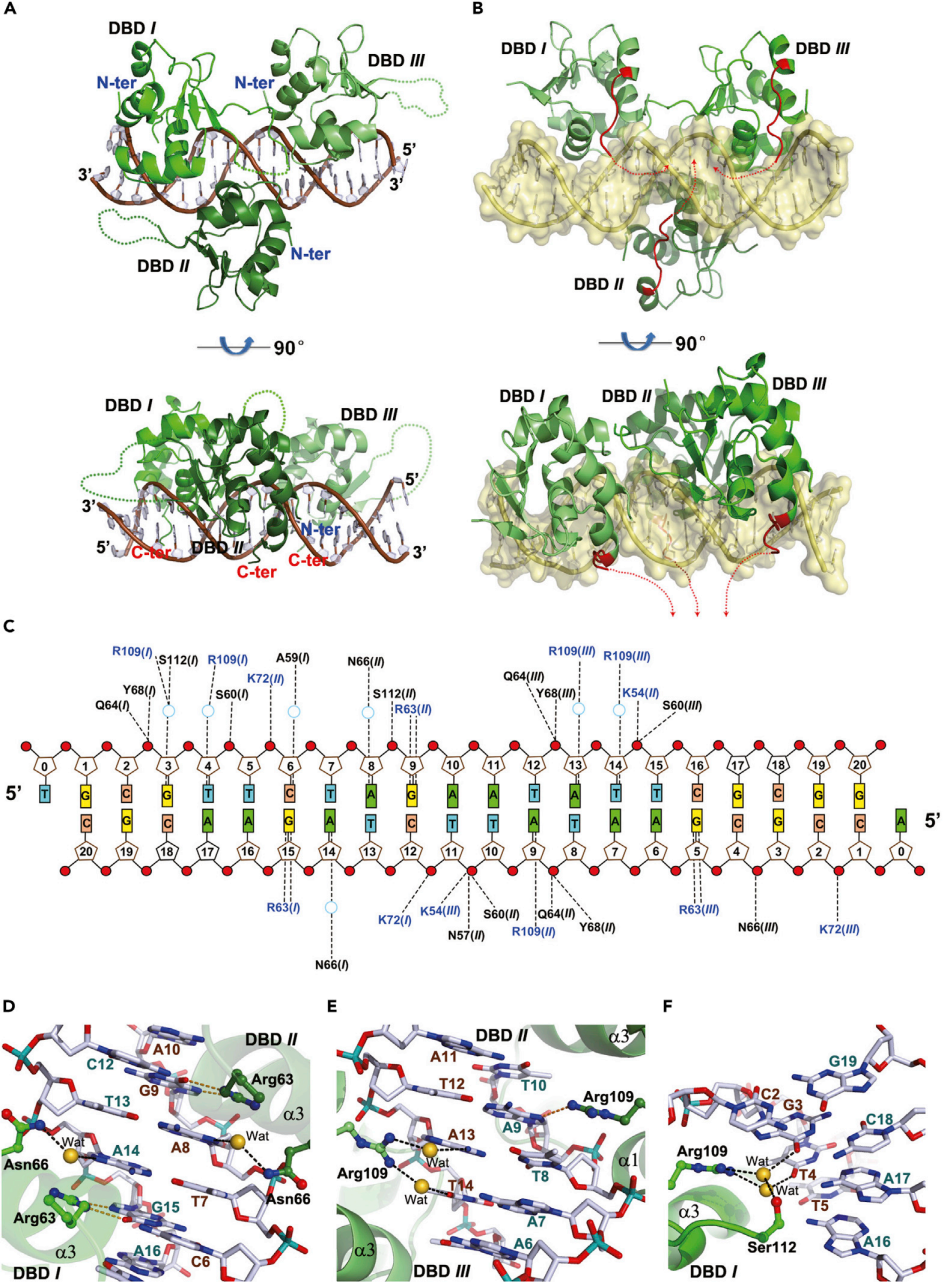
Structure of HSF2-DBD bound to cognate DNA comprising three binding sites (A) Overview of the HSF2-DBD trimer sitting on a 21-bp DNA duplex. (B) The same view highlighting the DNA-embracing model where the C-terminal peptides (red) of all DBDs extend to the opposite side of DNA and orient to the center of the protein trimer, which is presumably coordinated by an intermolecular coiled coil. (C) Schematic protein-DNA interactions present in the structure of HSF2 trimer bound to three-site HSE. (D) The bidentate hydrogen bond of Arg63 residue with the guanine in GAA, and a solvent-mediated hydrogen bond between Asn residue and the nucleobase preceding the GAA triplet. (E and F) The hydrogen bonds formed around Arg109 close to C terminus, which shows imperfect consistence between the three DBD copies. In (D–F), The direct and solvent-mediated hydrogen bonds are represented by orange and black dashed lines, respectively. The carbon atoms in protein and DNA are colored in green and gray, respectively.

In this structure, an HSF2 trimer binds to a tripartite HSE motif with one-to-one occupancy between the DBDs and the nGAAn repeats. All DBDs sit on one-half of DNA and are spatially arranged like an equilateral triangle seen from the top (Figures 2A and 2B). The two DBD copies located at the 5′ proximity (DBDs *I* and *II*) resemble the reported structure comprising a two-site tail-to-tail HSE (PDB: 5D8K) (Jaeger et al., 2016), whereas those located at the 3′ proximity (DBDs *II* and *III*) resemble the head-to-head dimer-binding structure determined in this study (Figure 1A). DBDs *I* and *III* that interact with the nGAAn repeats on the same DNA strand are almost identically oriented. By contrast, DBD *II* binding the middle repeat on the complementary strand is oppositely oriented.

As previously shown in the dimer-binding structures (Jaeger et al., 2016; Neudegger et al., 2016), the C terminus in each DBD takes an extended conformation toward the other side of DNA along the major groove, with the side chains of Lys110 and Ser112 hydrogen bonding to the phosphate backbone. These interactions greatly stabilize the linker preceding the trimerization domain and direct the coiled-coil standing on the opposite side of DNA. Noticeably, the C-terminal peptide of each DBD stretches toward the triangle center across the DNA axis (Figure 2B) and obviously positions the downstream coiled coil at an approximate 3-fold symmetry axis, which relates the three DBDs within a single trimer. This architecture may maximally coordinate HSF-HSE binding by generating roughly equivalent torque for each DBD.

Almost all sequence-specific interactions are conserved in this structure with those revealed in the dimer-binding structures (Figure 2C), including most direct and indirect hydrogen bonds from Arg63 and Asn66 (Figures 2D, S4A, and S4B) and the non-polar contacts around the methyl of thymines in TTC (Jaeger et al., 2016; Neudegger et al., 2016). Exceptionally, the side chain of Arg109 close to the C terminus in DBD *II* deeply inserts into the major groove and engages in a direct hydrogen bond with the purine preceding TTC (Figures 2E and S4C). This arginine in the other two DBDs, however, contacts two neighboring nucleobases by bifurcated solvent-mediated hydrogen bonds (Figures 2E, 2F, S4C and, S4D). In contrast to Asn66 in DBDs *I* and *II*, this residue in DBD *III* moves farther away from the major groove and engages in a hydrogen bond with the DNA backbone (Figure 2C).

### Structures of HSF1 trimer bound to two three-site HSEs

**Figure 3:**
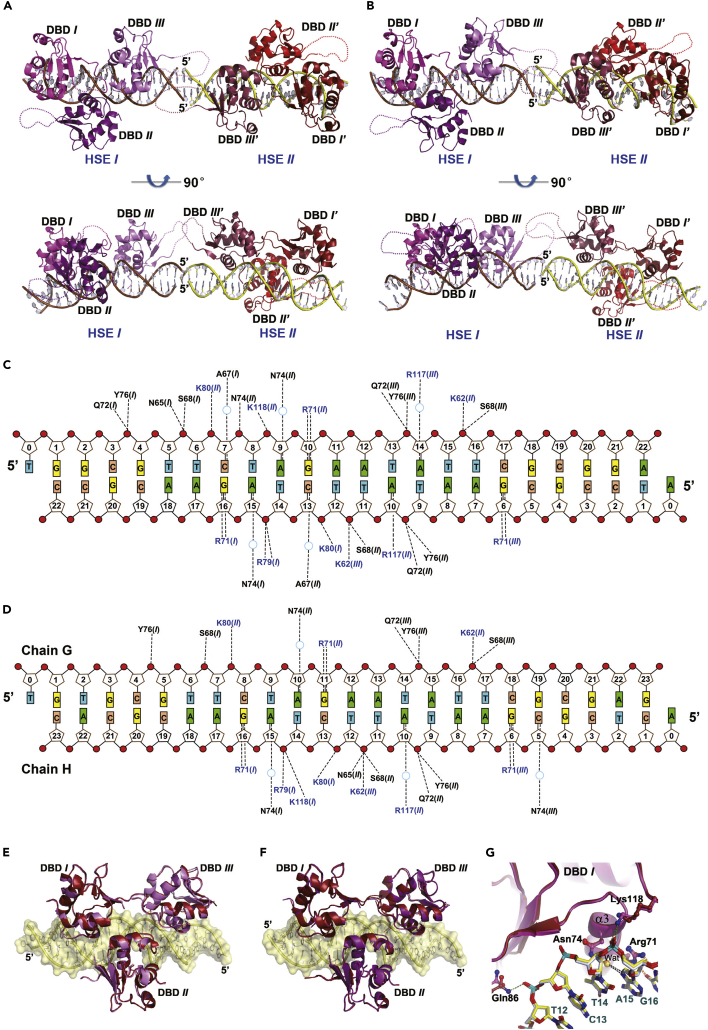
Structures of HSF1-DBD bound to cognate DNA comprising three binding sites (A and B) Overview of the HSF1-DBD trimers sitting on 23-bp (A) or 24-bp (B) DNA duplexes. In both structures two trimers reside in the asymmetric unit with different orientations. (C and D) Schematic protein-DNA interactions present in the structures comprising 23-bp (C) or 24-bp (D) DNA molecules. (E) Superimposition of HSF1 trimers (copy #1) between the two structures containing 23 (violet) and 24 bp (red) DNA molecules, where the DBDs in the two structures are colored in purple and blue, respectively. (F) Superimposition of the two HSF1 trimers present in the same asymmetric unit in the crystal structure containing 23-bp DNA molecules, where the DBDs bound to the two HSEs are colored in purple and red, respectively. (G) The hydrogen bonds formed along the major groove from the DBD occupying the HSE close to the 5′ end (DBD-*I*). The carbon atoms in protein and DNA are colored in violet and yellow, respectively.

Crystallization of the HSF1 trimer in complex with the same three-site HSE was unsuccessful until longer sticky-ended DNA duplexes were attempted. Two structures containing 23- and 24-bp oligonucleotides (Table S1) were determined and refined at 2.4 and 2.36 Å (Table 1). Different from the HSF2 structure, two HSF1 trimers bound to two intact HSEs reside in the asymmetric unit in both structures (Figures 3A and 3B), where the DNA duplexes were stacked in a head-to-head manner. As a consequence, the two HSF1 trimers are differently oriented with a large rotation angle between them (Figures 3A and 3B), but little conformational discrepancies exist among all the HSF1-HSE copies present in both structures (Table S3). The three DBD copies within either trimer are triangularly related similar to those present in the HSF2 structure (Figures 2A and 2B).

The majority of protein-DNA interactions are consistent with those revealed in the dimer-binding structures (Figures 3C and 3D). Like HSF2, Arg117 (corresponding to Arg109 in HSF2) in the middle DBD (DBD *II*) also inserts into the major groove and forms a direct hydrogen bond with the nucleobase 5′ to TTC. Slight interaction differences, however, were observed between the two trimers in each structure or between the two structures (Figures 3C, 3D, S5A, and S5B) attributable to trivial conformational discrepancies among the DBDs (Figures 3E and 3F). For example, in the structure comprising 23-bp DNA, the C terminus of DBD *II* in the first trimer is rigidified by a close contact between Lys118 and the phosphate backbone of DNA but not in the other two DBDs (Figure 3G). In contrast, such an electrostatic contact is observed in multiple DBDs in the other structure containing 24-bp DNA. Apparently, these subtle structural inconsistences are sequence independent but more likely dependent on local DNA conformation deviating from the standard B-form structure, which was likely driven by protein binding (Harteis and Schneider, 2014; Rohs et al., 2010).

The non-conserved cysteines Cys36 and Cys103 have been implicated in stress-induced HSF1 trimerization through intermolecular disulfide bond formation (Ahn and Thiele, 2003). In our structures, however, they are spatially distant from each other and pointing to opposite directions in each DBD itself and among different DBDs (Figure S5C). Owing to their exposure to solvent, they are in principle accessible for disulfide bond formation between trimers bound to an HSE comprising multiple binding sites.

### Structural determinants for auxiliary sequence-specificity interactions

The dinucleotide separating a pair of GAA triplets in the tail-to-tail arrangement is dominantly constrained to a pyrimidine-purine base-step according to ChIP-seq data compiled in the latest version of the JASPAR database (Figure S1A) (Khan et al., 2018), suggestive of its significance in sequence-specific interactions with HSFs, but the role of this dinucleotide was unclear. The structures determined in this study, however, provide a mechanistic explanation for the nucleotide preference at this position.

**Figure 4:**
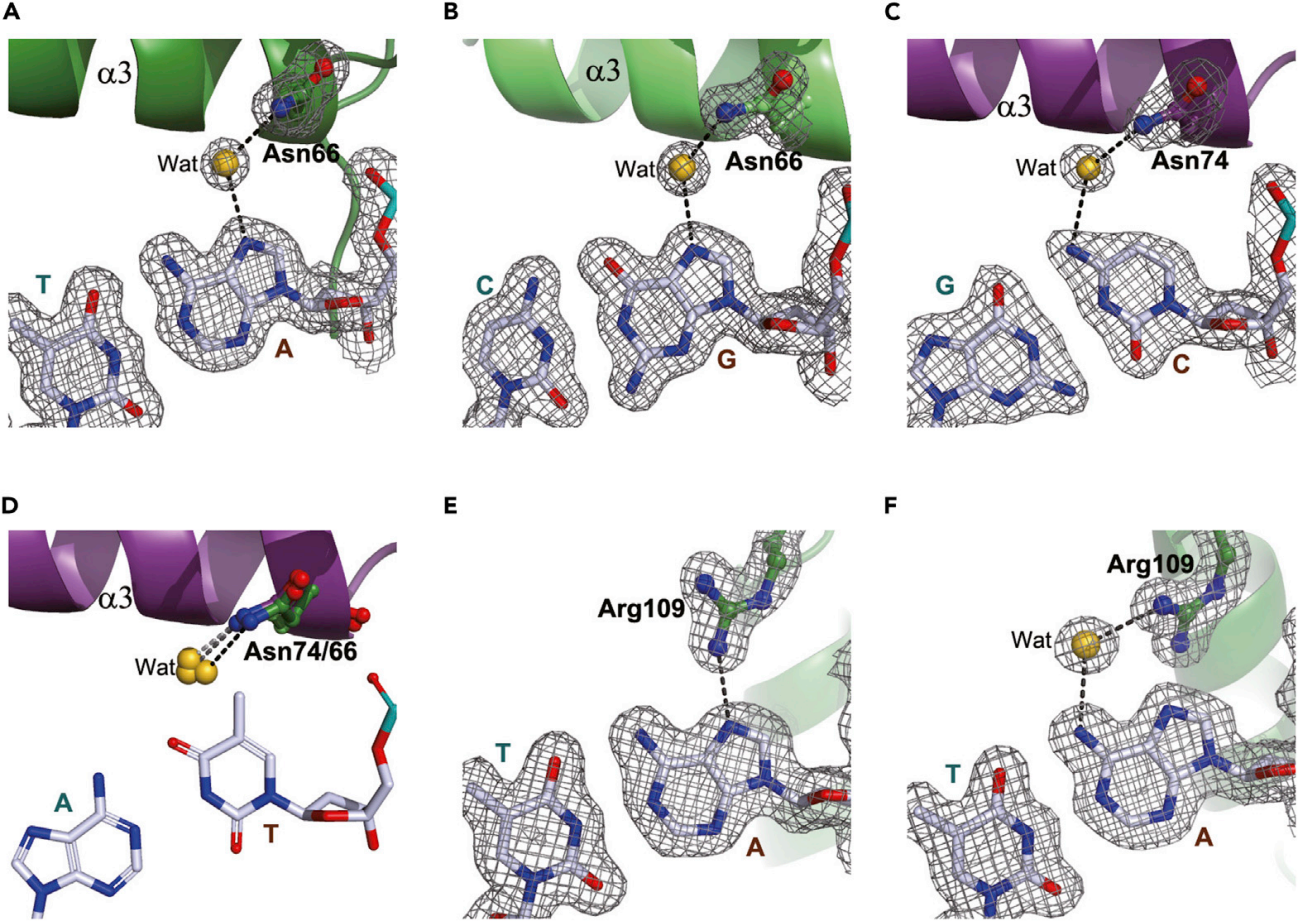
Hydrogen bonds formed around the dinucleobases separating the nGAAn repeats contributing auxiliary sequence specificity to the HSF-HSE interaction (A–C), The solvent-mediated hydrogen bonds between the imine group in adenine (A) or guanine (B) preceding GAA and Asn66 in HSF2, and that between the amino group in cytosine and Asn74 in HSF1 (C). (D), An artificial model replacing the cytosine shown in (C) with a thymine, the methyl group of which would sterically hinder the formation of hydrogen bonds from the conserved Asn residue. (E) The direct hydrogen bond formed between the imine group in an adenine preceding TTC and C-terminal Arg109 in the middle DBD (*II*). (F) The indirect hydrogen bond formed between the amino in an adenine preceding TTC and C-terminal Arg109 in the DBD close to the 3′-end (*III*). The 2*F*_o_ – *F*_c_ electron density contoured at 1.0 σ is shown as gray mesh.

In agreement with the reported tail-to-tail dimer-binding structures (Jaeger et al., 2016; Neudegger et al., 2016), an adenine preceding the GAA triplet is without exception contacted by a conserved asparagine (Asn66 in HSF2 or Asn74 in HSF1) present in the recognition helix through a solvent-mediated hydrogen bond in the three trimer-binding structures (Figure 4A). Similar contacts exist in the head-to-head double units, in which a guanine or a cytosine 5′-juxtaposed to GAA is hydrogen bonded with the same Asn residue (Figures 4B and 4C). Apart from a purine with an imino nitrogen participating in the contact, the amino nitrogen outside the pyrimidine ring in cytosine may also engage in an indirect hydrogen bond (Figure 4C), which, however, is only present in the structure of HSF1 bound to 24-bp DNA that shows more pronounced DNA bending than the other HSF1 structure (Figure 7F). Based on these observations, we reason that cytosine is a less favorable nucleobase than a purine at this position because its amino nitrogen is barely positioned at an indirect hydrogen bonding distance with the Asn residue only in a context with sufficient DNA backbone distortion. Furthermore, the presence of thymine at this position seems to be intolerable owing to steric hindrance from its methyl group, as shown in an artificial model with a C-T replacement (Figure 4D).

Dissimilar to the tail-to-tail arrangement, the dinucleotide separating a pair of head-to-head units is not constrained (Figure S1A) (Khan et al., 2018). In all the structures determined in this study, the adenine base preceding TTC is involved in either direct or indirect hydrogen bonding contacts with a conserved C-terminal arginine (Arg109 in HSF2 and Arg117 in HSF1) (Figures 4E and 4F). In contrast to the rigid Asn conformation contacting the nucleotide upstream of GAA (Figures 4A–4C), the variable side-chain rotamers of this Arg residue together with the flexible C-terminal peptide allows for higher tolerance of pyrimidine at this position.

### Synergic HSE binding mediated by the wing domain

**Figure 5:**
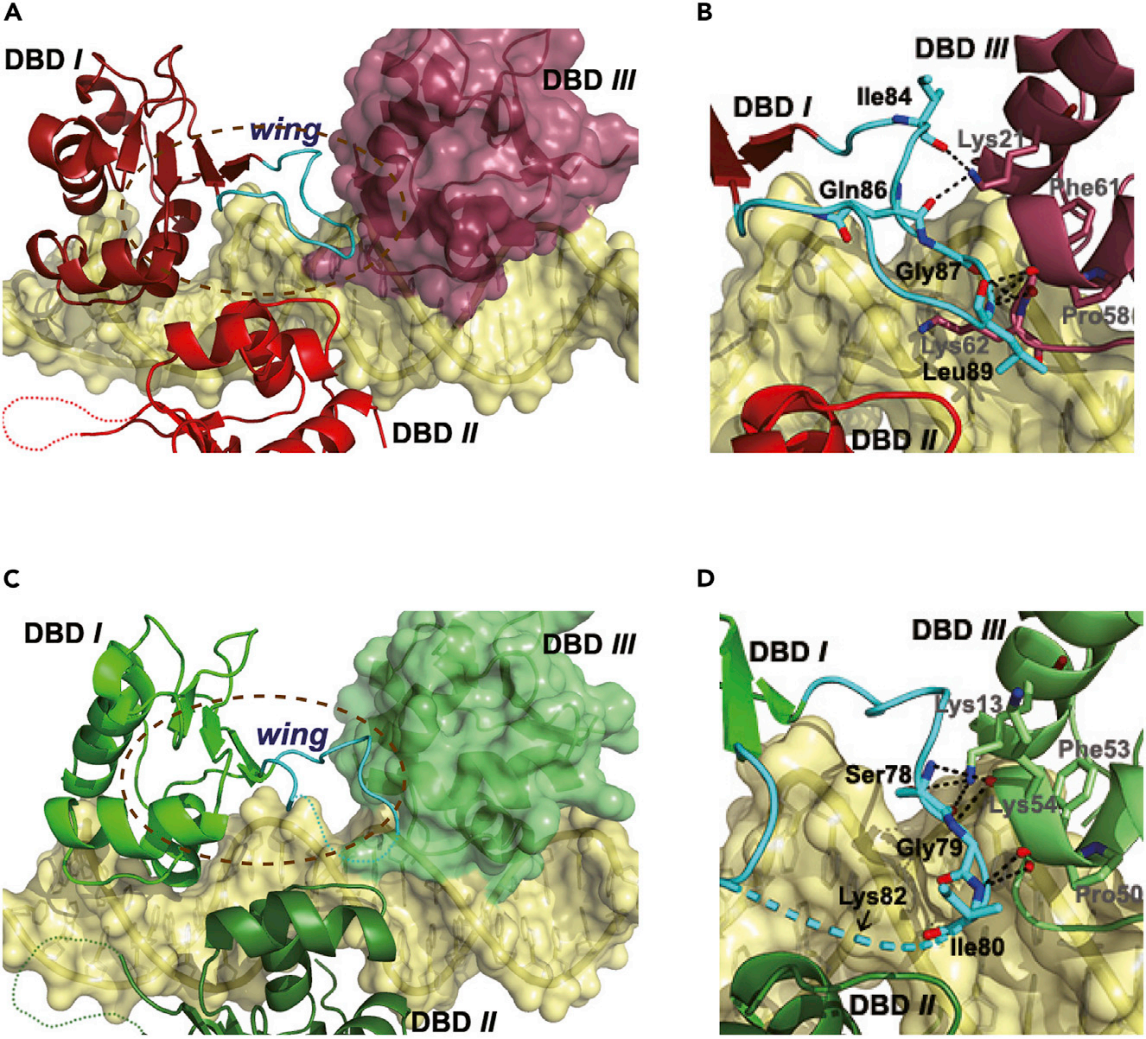
The wing domain mediating protein-protein interactions in HSF1/HSF2 trimers (A and C), The HSF1 (A) or HSF2 (C) wing loop in DBD *I* stretching toward DBD *III*, but not contacting DBD-*II*. (B and D), Close view of the ellipse-encircled area in (A) and (C), respectively, showing the contacts between DBD *I* and DBD *III*. HSF1 and HSF2 are colored in purple and green, respectively, whereas the loop domain is colored in cyan. In (B and D), the hydrogen bonds formed between DBDs are represented by dashed lines and in (D), the approximate position of Lys82, the potential SUMOylation site in HSF2, is indicated by an arrow.

The wing domain is characterized as a unique structural element in HSFs as it does not interact with DNA (Jaeger et al., 2016; Littlefield and Nelson, 1999; Neudegger et al., 2016), unlike most other transcription factors containing a similar motif (Harami et al., 2013). In our trimer-binding structures, the wing loop (residues 83–98 in HSF1 or 75–90 in HSF2) in DBDs *II* and *III* largely remained unstructured (Figures 2A, 3A, and 3B), but that in DBD *I* was well resolved with clear electron density for most amino acids therein. Particularly, the entire loop in an HSF1 trimer bound to the 23 bp DNA was visible and thus used for further analysis. This domain extends toward DBD *III* without contacting the DNA double helix or DBD *II* (Figure 5A). Its distal tip stretches into a shallow groove in DBD *III*, leading to generation of a hydrogen bond network at the DBD dimer interface (Figure 5B). Likewise, the wing domain in HSF2 DBD *I*, albeit not entirely visible in electron density, is engaged in a similar hydrogen bond network with DBD *III* (Figures 5C and 5D). Notably, all these contacts are formed between main-chain atoms except a lysine residue in helix α1 of DBD *III* (Lys21 in HSF1 or Lys13 in HSF2), indicating low interacting specificity between the two DBDs, which, however, very likely benefit for DBD interplays in an HSF1-HSF2 heterotrimer (Ostling et al., 2007; Sandqvist et al., 2009).

The wing-mediated interactions between DBDs *I* and *III* render this dimer interface area larger and more inter-DBD contacts than the other two (*I*-*II* and *II*-*III*) (Table S4). This observation is quite unexpected since these two DBDs bind to distal HSE repeats. The wing domains in DBDs *II* and *III*, albeit largely invisible, stretch outward to opposite directions and are apparently not involved in intramolecular contacts within this trimer (Figures 2A, 3A, and 3B) but are fully accessible for neighboring trimers bound to an HSE comprising multiple binding sites. Consequently, the wing domain may play important roles in synergizing the binding to HSEs by mediating both intra- and intermolecular interactions.

It is noteworthy that Lys82 in HSF2, an amino acid subject to SUMOylation (Anckar et al., 2006; Tateishi et al., 2009), is located very close to the dimer interface. The analyses based on monomer- and dimer-binding models have suggested that the highly flexible SUMO moiety adducted at this position may weaken protein-DNA interactions through dynamic steric interferences (Feng et al., 2016b; Tateishi et al., 2009). As a supportive evidence, our structures clearly indicate that SUMO conjugation in DBD *I* would utterly destroy the contacts to DBD *III* due to inevitable steric hindrance, whereas that occurring on DBD *II* or *III* may repress cooperative binding to a multiple-site HSE by interrupting trimer-trimer interactions. Despite not being a SUMOylation substrate (Becker et al., 2013; Jaeger et al., 2016; Tammsalu et al., 2014), binding of the wing domain in HSF1-DBD to replication protein A subunit 70 kDa (RPA70) has been reported, probably occurring at the distal region of the wing as well (Fujimoto et al., 2012). According to our structures, RPA70 binding may induce similar spatial conflicts among DBDs, which would result in disruption of protein-protein interactions, similar to the outcome of SUMOylation.

### A key amino acid position probably governing HSF-DNA binding specificity

**Figure 6:**
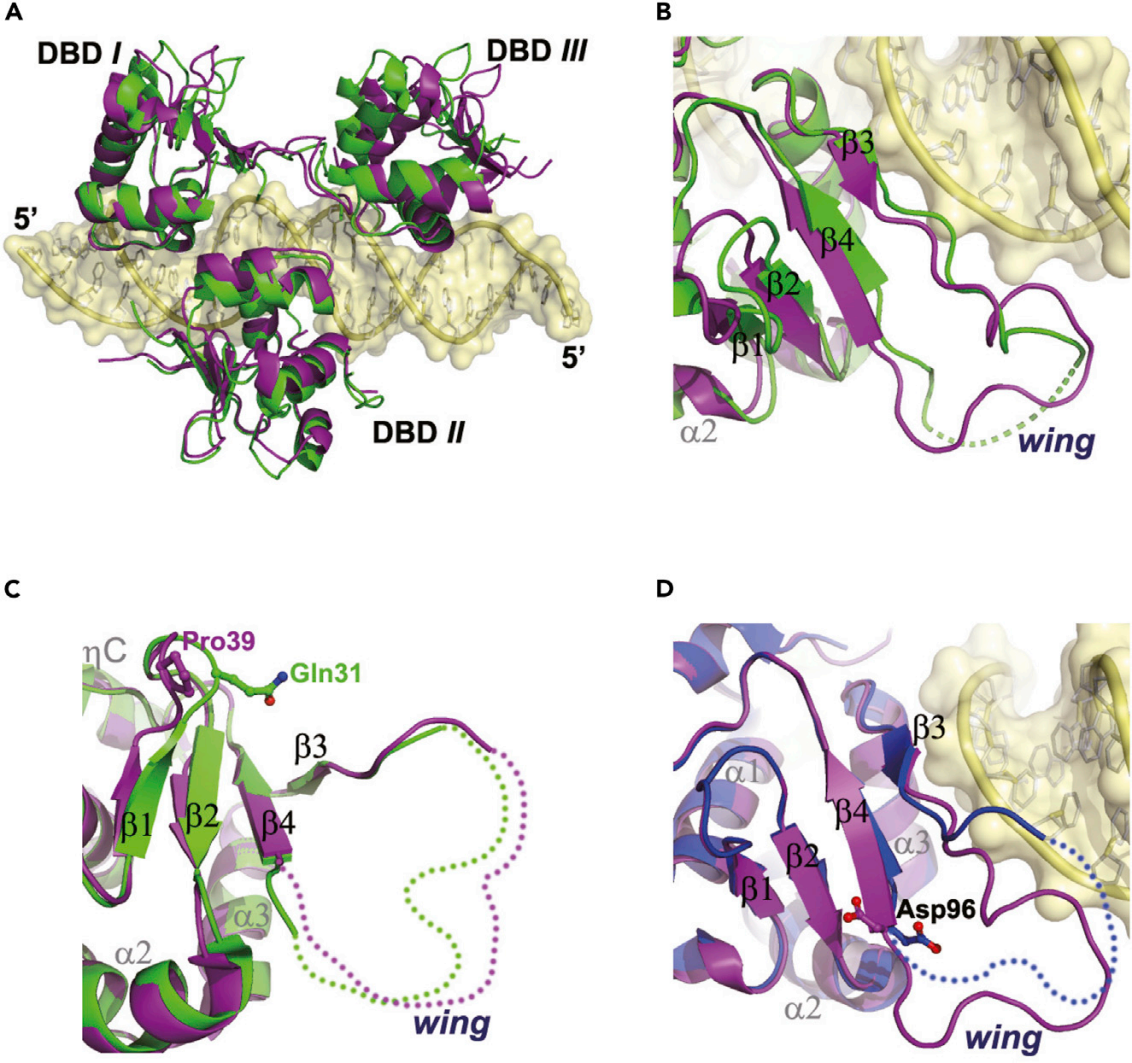
Structure comparison between HSF1 and HSF2 (A) Superimposition of HSF1 and HSF2 trimers. HSF1 and HSF2 are colored in purple and green, respectively. (B) Close view of the overlaid DBD *I* between HSF1 and HSF2. (C) Superimposition of DNA-free structures between HSF1-DBD (PDB: 5HDG) and HSF2-DBD (PDB: 5HDK). (D) Superimposition of HSF1 DNA-free structure (PDB: 5HDG) and DBD *I* in the trimer-binding structure comprising a 23-bp DNA duplex. In (A–C), HSF1 and HSF2 are colored in purple and green, respectively. In (D), the DNA-free structure is colored in blue.

It has been reported that mammalian HSFs possess differential specificity for different types of HSEs, although binding DNA in an almost identical manner (Kroeger and Morimoto, 1994; Yamamoto et al., 2009). Our structures revealed very similar protein-DNA interactions between HSF1 and HSF2, but the fact that they co-crystallized with DNA in different lengths and packing orientations seems to imply subtle discrepancies of binding specificity. To probe the structural determinants for this, we superimposed the two structures and noticed that the middle DBDs were well overlaid but the other two DBD counterparts were not. Both end-proximal DBDs (*I* and *III*) in HSF1 slightly translated along the DNA axis with respect to the HSF2 counterparts such that the HSF1 trimer occupied an approximately 2- to 3-bp longer DNA segment than HSF2 (Figure 6A).

When individual DBDs were superimposed, we observed some conformational variations, particularly in the β-sheet. Compared with HSF2, strands β2 and β4 in HSF1 are evidently longer and the shorter β1 strand is less twisted (Figure 6B). However, the overlaid DNA-free DBD structures (PDB: 5HDG and 5HDK) (Feng et al., 2016b) showed comparable length of β2 and β4 in both HSFs but noticeable difference in β1 twist (Figure 6C), which might be attributed to a non-conserved residue in the β1-β2 hairpin. The presence of a Pro residue at position 39 in HSF1 apparently constricts β1 to adopt a flatter conformation, while the corresponding Gln residue in HSF2 renders the hairpin more flexible and allows bigger twisting of β1.

Owing to the relatively poorer sequence conservation in relation to other regions in DBD, the wing domain shows distinct features among different HSFs. By comparison with the DNA-free structure (PDB: 5HDG), strands β2 and β4 of HSF1 is noticeably longer in the DNA-bound structures, indicating an occurrence of β-strand extension induced by HSF-HSE binding (Figures 6D and S6). Thus, an intriguing question is why this conformational change occurs in HSF1 but not in HSF2. We observed that two residues located at the wing C terminus in HSF1, which are not conserved in HSF2, changed from loop conformation to β-strand engagement and thus lengthening strand β4 from its N terminus; in particular Asp96 therein underwent a dramatic conformational change by shifting its negatively charged side chain from DNA accessible to an orientation against the protein interior (Figures 6D and S6). Very likely, this shift, attributable to the same charge repulsion from the phosphate backbone of DNA, prominently restrains the motional freedom of the wing loop. The corresponding position in HSF2, however, is a Gly-Pro dipeptide (Figure S1B), which renders the loop highly flexible and probably serves as a prerequisite for the SUMOylation on neighboring Lys82 (Anckar et al., 2006). These distinct features, which determine the relative flexibility of the wing domain, may be closely linked with the HSF-target specificity. In principle an elastic wing favors compact trimer formation of HSF2, whereas on the contrary, the less flexible one in HSF1 only allows for the formation of a more extended trimer upon DNA binding.

### Local variations in DNA structure provide higher resolution specificity distinguishing HSF1 and HSF2

As sequence-specific transcription factors, HSFs interact with HSEs through a set of base-specific hydrogen bonds formed in the major groove. This direct readout mechanism, however, is insufficient to account for the differential specificity among HSF family members comprising homologous DBDs and almost identical protein-DNA interactions. Since DNA-shape readout is often exploited for higher resolution specificity to distinguish DNA-binding proteins within the same family (Privalov et al., 2007; Rohs et al., 2010), we analyzed DNA topography in our structures and the published ones comprising tail-to-tail two-site HSEs (PDB: 5D8K and 5D5U) (Jaeger et al., 2016; Neudegger et al., 2016), to see if this rule applies to the HSF family.

The geometric parameters calculated from program *3DNA* (Lu and Olson, 2008) revealed marked major groove widening in the dimer-binding structures comprising a tail-to-tail HSE-motif under both circumstances with HSF1 and HSF2 binding (Figures S7A and S7B). However, major groove widening was not observed in the structures comprising head-to-head counterparts. In good consistence, maximal widening occurs in the middle of the first two inverted repeats in the three-site HSEs, indicating that the dinucleotide separating a TTC triplet from a following GAA triplet is the primary position where the major groove is broadened. Conversely, marked minor groove narrowing occurs in the head-to-head rather than the tail-to-tail dimer-bound structures. Dissimilar to the major groove widening, however, comparable extent of minor groove narrowing was observed at the dinucleotide bridging both TTC-GAA and GAA-TTC dual triplets in either HS1 or HSF2 trimer-binding structures (Figures S7C and S7D). This analysis undoubtedly reveals that subtle local DNA shape deformations from the standard B-form conformation are present in these structures, which seem to be sequence dependent and likely driven by HSF binding.

**Figure 7:**
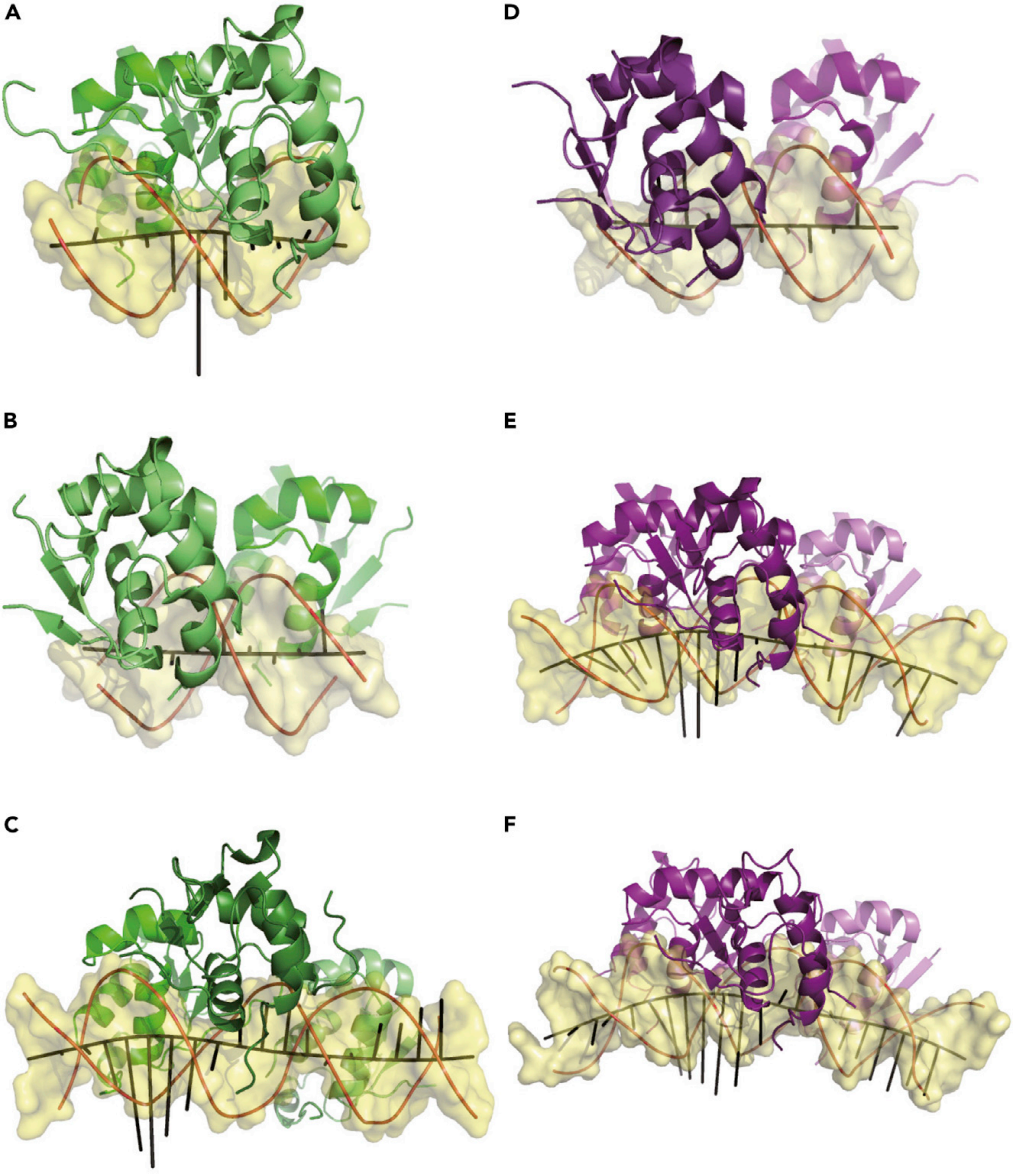
DNA curvature calculated using *Curves+* (A) Structure of HSF2 bound to two-site HSE in tail-to-tail orientation (PDB: 5D8K). (B) Structure of HSF2 bound to two-site HSE in head-to-head orientation. (C) Structure of HSF2 bound to three-site HSE. (D) Structure of HSF1 bound to two-site HSE in head-to-head orientation (E) Structure of HSF1 bound to three-site HSE (23 bp). (F) Structure of HSF1 bound to three-site HSE (24 bp). Curvature of individual base pairs is represented by black bars vertical to the axis of the DNA duplex, the longer a bar, the greater the curvature at the corresponding base pair step.

Curvature of the DNA double helix was analyzed using *Curves*+ (Blanchet et al., 2011). By showing the curvature denotation (the black bars in Figure 7), a slight bend to the opposite direction of HSF binding is visible at the middle of the tail-to-tail dual motifs (Figure 7A) but not in the head-to-head counterparts (Figures 7B and 7D), which well concerts with the major groove widening. In the HSF2 trimer-binding structure, however, obvious bending is observed at the 5′-proximal TTC triplet bound by DBD *I* rather than the downstream purine-pyrimidine base pair, while the DNA at the other two binding sites remains a relatively linear shape (Figure 7C). By contrast, in both HSF1 trimer-binding structures, dramatic bending occurs at both the 5′-proximal HSE triplet and the downstream dinucleotide, although relatively smaller bending occurs at the 3′-proximal nGAAn repeat bound by DBD *III*. As a net effect, the overall DNA bending in the HSF1 structures is 25°–29° (Figures 7E and 7F), much larger than the 10° bending in HSF2 structure (Figure 7C).

Conclusively, HSF1 and HSF2 both induce local alterations in DNA conformation during HSE binding with considerable difference in DNA curvature. Apparently, HSF1 binding drives significantly larger DNA bending than HSF2 binding, likely establishing an indirect readout mechanism that provides higher resolution for the HSE binding specificity distinguishing the two closely related HSFs.

## Discussion

The trimer-binding structures presented in this study depict a picture partially reflecting the physiological assembly of the HSF-HSE complex, in which the DBDs bind HSEs in a triangular arrangement and anchors the downstream coiled coil above the center of the triangle. This trimeric architecture forces the coiled coil to serve as a pseudo-3-fold symmetry axis to relate the three DBDs across the DNA duplex (Figure 2B). When bound to three binding sites that are linearly arranged, such intramolecular symmetry would in principle maximize the synergetic avidity by generating almost equal torque to individual DBDs and thus avoid imbalanced protein-DNA interactions. In another respect, this spatial arrangement allows each DBD to have almost the same chance to receive regulatory inputs, enabling complex transcriptional regulation in various cellular contexts.

Different from most DNA-binding proteins, the wing domain in HSFs does not contact DNA and hence is surmised to be involved in protein-protein interactions (Ahn et al., 2001; Cicero et al., 2001; Jaeger et al., 2016; Littlefield and Nelson, 1999; Neudegger et al., 2016). However, no direct evidence has been provided from previous structural studies. Our structures, binding as trimers, revealed such interactions. We note in particular the extensive interdomain contacts formed between the two DBDs located at discontinued binding sites, which seem to be necessary for sufficient avidity by effectively stabilizing the DNA-bound trimeric architecture (Figure 5). It is also noteworthy that these contacts are sequence independent owing to the exclusion of hydrogen bonding between side chains, which would in principle provide good tolerance for HSF1/2 interplay within a heterotrimer. Besides the intramolecular contacts, our structures also prompt that the wing domain may engage in the interactions between HSF trimers bound to a long array of the nGAAn repeats. All these observations solidly indicate the importance of the wing domain in synergizing HSE binding, which agrees well with the conclusion drawn from biochemical studies (Ahn et al., 2001; Cicero et al., 2001).

A plethora of experimental data has demonstrated that mammalian HSFs exhibit differential specificity for target recognition, although they share a highly conserved DNA-binding domain and very similar protein-DNA interactions (Figures 2 and 3) (Kroeger and Morimoto, 1994; Yamamoto et al., 2009). Mechanistic understanding for this differentiation was missing, but fortunately the complex structures determined in this study allowed us to perform a systematic analysis for addressing this question. We found that, in particular, two properties might contribute to the divergence between HSF1 and HSF2. First, owing to β-strand elongation induced by DNA binding (Figure 6B), the wing domain in HSF1 is relatively less flexible than that in HSF2, which results in slightly different quaternary conformations between them (Figure 6A). Second, the DNA conformational variations that were not fully appreciated previously may serve as a *trans* driving force to impact the differential specificity of the two HSFs. A further interesting observation is the significant divergence in DNA curvature between the HSF1 and HSF2 structures (Figure 7), which strongly suggests that HSF1 prefers to recognize and bind a more curved DNA than HSF2. Such a shape readout mechanism may well account for the fact that HSF1 prefers long arrays of the inverted nGAAn units but HSF2 rather prefers short arrays (Kroeger and Morimoto, 1994), because in principle bending of a longer segment covered by protein binding is energetically less expensive than a shorter one (Harteis and Schneider, 2014). In this sense, more complex conformational variations in DNA are expected in the case of gene transcription fine-tuning by an HSF1/HSF2 heterotrimer.

### Limitations of the study

There are two limitations to this study: one is that, apart from the DBD, other domains in HSF showing poor crystallizability are not present in our crystal structures, and the other is that our conclusions are drawn solely based on structural analysis and therefore further experimental verifications from *in vitro* and *in vivo* studies with the full-length HSFs will be needed in the future.

## STAR★Methods

### Key resources table

**Table:** 

REAGENT or RESOURCE	SOURCE	IDENTIFIER
**Bacterial and virus strains**		

*Escherichia coli*: DH5α	Biomed	Cat# BC102-01
*Escherichia coli*: Rosetta2	Novagen	Cat# 71397-3
Chemicals, peptides, and recombinant proteins		
Isopropyl b-D-thiogalactopyranoside (IPTG)	Sangon Biotech	Cat# A600168-0025
1,4-Dithio-DL-threitol (DTT)	Sangon Biotech	Cat# A100281-0005

**Critical commercial assays**		

Index Kit	Hampton Research	Cat# HR2-144
CrystalScreen 1/2 Kit	Hampton Research	Cat# HR2-110; HR2-112
PEG Rx 1/2 Kit	Hampton Research	Cat# HR2-082; HR2-084
PEG/Ion 1/2 Screen Kit	Hampton Research	Cat# HR2-126; HR2-098

**Deposited data**		

Structure of HSF2-HtH HSE	This study	PDB: 7DCI
Structure of HSF1-HtH HSE	This study	PDB: 7DCJ
Structure of HSF2-3site HSE	This study	PDB: 7DCU
Structure of HSF1-3site HSE #1	This study	PDB: 7DCS
Structure of HSF1-3site HSE #2	This study	PDB: 7DCT

**Oligonucleotides**		

12 bp HtH HSE: GGGAATATTCCC	Sangon Biotech	N/A
14 bp HtH HSE: GCCGAATATTCGGC	Sangon Biotech	N/A
21 bp HSE_F: ACCGCGAATATTCTAGAACGC	Invitrogen	N/A
21 bp HSE_R: TGCGTTCTAGAATATTCGCGG	Invitrogen	N/A
23 bp HSE_F: ATCCGCGAATATTCTAGAACGCC	Invitrogen	N/A
23 bp HSE_R: TGGCGTTCTAGAATATTCGCGGA	Invitrogen	N/A
24 bp HSE_F: ACTCGCGAATATTCTAGAACGCAC	Invitrogen	N/A
24 bp HSE_R: TGTGCGTTCTAGAATATTCGCGAG	Invitrogen	N/A

**Recombinant DNA**		

Plasmid: pMAL-c4e	NEB	Cat# N8105
Plasmid: pRK603	Addgene	Cat# 8831
Plasmid: pMAL-c4e-HSF1 15-122	This study	N/A
Plasmid: pMAL-c4e-HSF2 7-115	This study	N/A

**Software and algorithms**		

HLK2000	Otwinowski and Minor, 1997	https://www.hkl-xray.com/hkl-2000
iMosflm	Battye et al., 2011	https://www.mrc-lmb.cam.ac.uk/mosflm/mosflm/
CCP4	Winn et al., 2011	http://www.ccp4.ac.uk/
PHENIX	Adams et al., 2010	https://www.phenix-online.org
Coot	Emsley et al., 2010	https://www2.mrc-lmb.cam.ac.uk/personal/pemsley/coot/
PyMOL	Schrodinger, 2010	https://pymol.org/2/
STARANISO & autoBUSTER	Bricogne et al., 2017	http://staraniso.globalphasing.org
Nucplot	Luscombe et al., 1997	https://www.ebi.ac.uk/thornton-srv/software/NUCPLOT/
3DNA	Lu and Olson, 2008	http://www.guptalab.org/3dna/
Curves+	Lavery et al., 2009	http://curvesplus.bsc.es/
PDBePISA	Krissinel and Henrick, 2007	https://www.ebi.ac.uk/pdbe/pisa/

### Resource availability

#### Lead contact

Further information and requests for resources and reagents should be directed to and will be fulfilled by the lead contact, Jingjin Ding (jding@ibp.ac.cn).

#### Materials availability

All unique/stable reagents generated in this study are available from the Lead Contact without restriction.

### Method details

#### Protein expression and purification

Expression and purification of the DNA-binding domain (DBD) in HSF1 (residues 15-120) and HSF2 (residues 7-112) were conducted following a previously established protocol (Feng et al., 2016a, 2016b). In brief, the corresponding nucleotide sequences were inserted into a pMAL-c4E plasmid for production of a destination protein with its N-terminal end fused with maltose-binding protein (MBP). A Tobacco Etch Virus (TEV) protease cleavage site and a His6-tag were introduced between MBP and the DBDs by PCR amplification. A modified *E*. *coli* strain used for protein expression was generated by transforming the plasmid pRK603 (Kapust and Waugh, 2000), which contains a gene encoding the TEV protease, into Rosetta2(DE3) before competent cells were made. The recombinant fusion protein was produced in this strain at 30°C with an incubation for 4 h after the induction with 0.3 mM isopropyl β-D-1-thiogalactopyranoside (IPTG). Immediately after that, expression of the TEV protease was induced by addition of anhydrotetracyclin hydrochloride (aTet) at a final concentration of 100 ng/ml for another 2 h at 30°C, during which the MBP-tag was removed *in vivo* from the fusion protein. Harvested cells were lysed using a high-pressure crusher at 4°C, and the supernatant was immediately loaded onto a Ni2+-NTA chromatography column (Novagen), followed by column washing with 40 mM imidazole and elution with 250 mM imidazole added in the lysis buffer. The protein was further purified by cation-exchange chromatography using a Hitrap SP HP 5 ml column (GE Healthcare) and size-exclusion chromatography using a HiLoad 16/600 Superdex 75 column (GE Healthcare) equilibrated with a buffer containing 20 mM Tris, pH 8.0, 150 mM NaCl, 1 mM DTT, 0.2 mM EDTA. The purified protein was concentrated to 40 mg/ml (measured at OD280) and store at -80°C.

#### Protein crystallization

A number of nucleotide sequences with different lengths and compositions were attempted in co-crystallization with HSF1/2-DBDs, and those leading to structure determination of the desired protein-DNA complexes are listed in Table S1. DNA duplexes were formed from pairs of complementary oligonucleotides by annealing in TE buffer at a concentration of 3 mM. In crystallization trails of the DBDs with 2-site HSEs, the complexes were formed by mixing protein and DNA with a molar ratio at 2:1.5, while in co-crystallization of DBDs with DNA containing 3-site HSEs, a protein/DNA molar ratio of 3:1.2 were used. No further purifications were conducted on the mixed samples. Initial crystallization conditions were screened with 10 commercial kits from Hampton Research in sitting-drop setups using a Mosquito crystallization robot (TTP Labtech). Subsequent optimizations were performed using hanging-drop vapor diffusion method by hand at 20°C. Crystals grown under optimal conditions, which are summarized in Table S2, were obtained from mixture of 1 μl sample and 1 μl reservoir solution.

#### Diffraction data collection and processing

The crystals used for X-ray data collection were presoaked in a cryoprotectant (100% paraffin oil for DBDs bound to 2-site HSEs or the reservoir supplemented with 10% (w/v) sucrose for DBDs bound to 3-site HSEs) for 10 s before flash cooling in streams of liquid nitrogen. All diffraction data sets were collected on beamlines BL17U1 (Wang et al., 2018) and BL18U1 with different wavelengths at Shanghai Synchrotron Radiation Facility (SSRF), China. All data sets except that for HSF1-DBD with 2-site HSE, which was processed using *HKL*2000 (Otwinowski and Minor, 1997), were indexed, integrated using *iMosflm* (Battye et al., 2011) and scaled using Scala from the *CCP*4 program suite (Winn et al., 2011).

#### Structure determination and refinement

All structures were determined by means of molecular replacement using *Phaser* (Bunkoczi et al., 2013). Two DNA-free structures recently solved in our lab, HSF1-DBD (PDB entry 5HDG) and HSF2-DBD (PDB entry 5HDK) (Feng et al., 2016b), together with the standard B-form DNA duplexes generated using *Coot* (Emsley et al., 2010) were used as search models for phasing. After density modification and automatic model building using the *PHENIX* program suite (Adams et al., 2010), the resultant models were refined using *phenix*.*refine* with several rounds of manual remodeling in *Coot* (Emsley et al., 2010) between refinement cycles. The diffraction data for both structures of HSF1-DBD bound to a 3-site HSE, 23 bp or 24 bp containing a 1-nt 5’-overhang, showed severe anisotropy and resultantly gave rise to unreasonably high R-factors after refinement. To tackle this problem, an anisotropy diffraction cut-off was done on the *STARANISO* webserver (http://staraniso.globalphasing.org) using Bayesian estimation of structure amplitudes. The output MTZ file was used in subsequent refinement using *autoBUSTER* (Bricogne et al., 2017) and *phaser*.*refine* (Adams et al., 2010). Statistics of data collection and structure refinement was summarized in Table 1.

#### Structure analysis

The final refined models were validated using *MolProbity* (Chen et al., 2010). Statistics of data collection and structure refinement was summarized in Table 1. All figures displaying structure representations were prepared using the molecular-visualization program *Pymol* (Schrodinger, 2010). Protein-DNA interactions were analyzed and schematically depicted using *Nucplot* (Luscombe et al., 1997), while the contacts between DBDs were analyzed using the *PDBePISA* server at European Bioinformatics Institute (Krissinel and Henrick, 2007). Stereochemical parameters of DNA such as roll angles, groove widths and backbone curvature were obtained using 3*DNA* (Lu and Olson, 2008) and the *Curves*+ websever (Lavery et al., 2009).

### Quantification and statistical analysis

This manuscript does not include quantification or statistical analysis.

## Data Availability

• The coordinates and structure factors generated during this study have been deposited in the Protein Data Bank under the accession codes 7DCI (HSF2-DBD in complex with 2-site head-to-head HSE), 7DCJ (HSF1-DBD in complex with 2-site head-to-head HSE), 7DCU (HSF2-DBD in complex with 3-site HSE), 7DCS (HSF1-DBD in complex with 3-site HSE, 23 bp), and 7DCT (HSF1-DBD in complex with 3-site HSE, 24 bp). • This paper does not report original code. • Any additional information required to reanalyze the data reported in this paper is available from the lead contact upon request.

## References

[bib1] Adams P.D., Afonine P.V., Bunkoczi G., Chen V.B., Davis I.W., Echols N., Headd J.J., Hung L.W., Kapral G.J., Grosse-Kunstleve R.W. (2010). PHENIX: a comprehensive Python-based system for macromolecular structure solution. Acta Crystallogr. Section D, Biol. Crystallogr..

[bib2] Ahn S.G., Liu P.C., Klyachko K., Morimoto R.I., Thiele D.J. (2001). The loop domain of heat shock transcription factor 1 dictates DNA-binding specificity and responses to heat stress. Genes Dev..

[bib3] Ahn S.G., Thiele D.J. (2003). Redox regulation of mammalian heat shock factor 1 is essential for Hsp gene activation and protection from stress. Genes Dev..

[bib4] Akerfelt M., Morimoto R.I., Sistonen L. (2010). Heat shock factors: integrators of cell stress, development and lifespan. Nat. Rev. Mol. Cell Biol..

[bib5] Akerfelt M., Vihervaara A., Laiho A., Conter A., Christians E.S., Sistonen L., Henriksson E. (2010). Heat shock transcription factor 1 localizes to sex chromatin during meiotic repression. J. Biol. Chem..

[bib6] Amin J., Ananthan J., Voellmy R. (1988). Key features of heat shock regulatory elements. Mol. Cell Biol..

[bib7] Anckar J., Hietakangas V., Denessiouk K., Thiele D.J., Johnson M.S., Sistonen L. (2006). Inhibition of DNA binding by differential sumoylation of heat shock factors. Mol. Cell Biol..

[bib8] Battye T.G., Kontogiannis L., Johnson O., Powell H.R., Leslie A.G. (2011). iMOSFLM: a new graphical interface for diffraction-image processing with MOSFLM. Acta Crystallogr. Section D Biol. Crystallogr..

[bib9] Becker J., Barysch S.V., Karaca S., Dittner C., Hsiao H.H., Berriel Diaz M., Herzig S., Urlaub H., Melchior F. (2013). Detecting endogenous SUMO targets in mammalian cells and tissues. Nat. Struct. Mol. Biol..

[bib10] Bjork J.K., Sistonen L. (2010). Regulation of the members of the mammalian heat shock factor family. FEBS J..

[bib11] Blanchet C., Pasi M., Zakrzewska K., Lavery R. (2011). CURVES+ web server for analyzing and visualizing the helical, backbone and groove parameters of nucleic acid structures. Nucleic Acids Res..

[bib12] Bricogne G., Blanc E., Brandl M., Flensburg C., Keller P., Paciorek W., Roversi P., Sharff A., Smart O.S., Vonrhein C. (2017). BUSTER.

[bib13] Bunkoczi G., Echols N., McCoy A.J., Oeffner R.D., Adams P.D., Read R.J. (2013). Phaser.MRage: automated molecular replacement. Acta Crystallogr. D Biol. Crystallogr..

[bib14] Chen V.B., Arendall W.B., Headd J.J., Keedy D.A., Immormino R.M., Kapral G.J., Murray L.W., Richardson J.S., Richardson D.C. (2010). MolProbity: all-atom structure validation for macromolecular crystallography. Acta Crystallogr. D Biol. Crystallogr..

[bib15] Cicero M.P., Hubl S.T., Harrison C.J., Littlefield O., Hardy J.A., Nelson H.C. (2001). The wing in yeast heat shock transcription factor (HSF) DNA-binding domain is required for full activity. Nucleic Acids Res..

[bib16] Dai C., Whitesell L., Rogers A.B., Lindquist S. (2007). Heat shock factor 1 is a powerful multifaceted modifier of carcinogenesis. Cell.

[bib17] Emsley P., Lohkamp B., Scott W.G., Cowtan K. (2010). Features and development of Coot. Acta Crystallogr. D Biol. Crystallogr..

[bib18] Feng H., Liu W., Wang da C. (2016). Purification, crystallization and X-ray diffraction analysis of the DNA-binding domain of human heat-shock factor 2. Acta Crystallogr. F Struct. Biol. Commun..

[bib19] Feng H., Wang S., Guo L., Punekar A.S., Ladenstein R., Wang D.C., Liu W. (2016). MD simulation of high-resolution X-ray structures reveals post-translational modification dependent conformational changes in HSF-DNA interaction. Protein Cell.

[bib20] Fujimoto M., Takaki E., Takii R., Tan K., Prakasam R., Hayashida N., Iemura S., Natsume T., Nakai A. (2012). RPA assists HSF1 access to nucleosomal DNA by recruiting histone chaperone FACT. Mol. Cell.

[bib21] Gidalevitz T., Kikis E.A., Morimoto R.I. (2010). A cellular perspective on conformational disease: the role of genetic background and proteostasis networks. Curr. Opin. Struct. Biol..

[bib22] Gomez-Pastor R., Burchfiel E.T., Thiele D.J. (2018). Regulation of heat shock transcription factors and their roles in physiology and disease. Nat. Rev. Mol. Cell Biol..

[bib23] Guertin M.J., Lis J.T. (2010). Chromatin landscape dictates HSF binding to target DNA elements. PLoS Genet..

[bib24] Guisbert E., Czyz D.M., Richter K., McMullen P.D., Morimoto R.I. (2013). Identification of a tissue-selective heat shock response regulatory network. PLoS Genet..

[bib25] Harami G.M., Gyimesi M., Kovacs M. (2013). From keys to bulldozers: expanding roles for winged helix domains in nucleic-acid-binding proteins. Trends Biochem. Sci..

[bib26] Harteis S., Schneider S. (2014). Making the bend: DNA tertiary structure and protein-DNA interactions. Int. J. Mol. Sci..

[bib27] Jaeger A.M., Pemble C.W.t., Sistonen L., Thiele D.J. (2016). Structures of HSF2 reveal mechanisms for differential regulation of human heat-shock factors. Nat. Struct. Mol. Biol..

[bib28] Kapust R.B., Waugh D.S. (2000). Controlled intracellular processing of fusion proteins by TEV protease. Protein Expr. Purif..

[bib29] Khan A., Fornes O., Stigliani A., Gheorghe M., Castro-Mondragon J.A., van der Lee R., Bessy A., Cheneby J., Kulkarni S.R., Tan G. (2018). Jaspar 2018: update of the open-access database of transcription factor binding profiles and its web framework. Nucleic Acids Res..

[bib30] Kline M.P., Morimoto R.I. (1997). Repression of the heat shock factor 1 transcriptional activation domain is modulated by constitutive phosphorylation. Mol. Cell Biol..

[bib31] Krissinel E., Henrick K. (2007). Inference of macromolecular assemblies from crystalline state. J. Mol. Biol..

[bib32] Kroeger P.E., Morimoto R.I. (1994). Selection of new HSF1 and HSF2 DNA-binding sites reveals difference in trimer cooperativity. Mol. Cell Biol.

[bib33] Lavery R., Moakher M., Maddocks J.H., Petkeviciute D., Zakrzewska K. (2009). Conformational analysis of nucleic acids revisited: Curves+. Nucleic Acids Res..

[bib34] Littlefield O., Nelson H.C. (1999). A new use for the 'wing' of the 'winged' helix-turn-helix motif in the HSF-DNA cocrystal. Nat. Struct. Biol..

[bib35] Lu X.J., Olson W.K. (2008). 3DNA: a versatile, integrated software system for the analysis, rebuilding and visualization of three-dimensional nucleic-acid structures. Nat. Protoc..

[bib36] Luscombe N.M., Laskowski R.A., Thornton J.M. (1997). NUCPLOT: a program to generate schematic diagrams of protein-nucleic acid interactions. Nucleic Acids Res..

[bib37] Neef D.W., Jaeger A.M., Thiele D.J. (2011). Heat shock transcription factor 1 as a therapeutic target in neurodegenerative diseases. Nat. Rev. Drug Discov..

[bib38] Neudegger T., Verghese J., Hayer-Hartl M., Hartl F.U., Bracher A. (2016). Structure of human heat-shock transcription factor 1 in complex with DNA. Nat. Struct. Mol. Biol..

[bib39] Ostling P., Bjork J.K., Roos-Mattjus P., Mezger V., Sistonen L. (2007). Heat shock factor 2 (HSF2) contributes to inducible expression of hsp genes through interplay with HSF1. J. Biol. Chem..

[bib40] Otwinowski Z., Minor W. (1997). Processing of X-ray diffraction data collected in oscillation mode. Methods Enzymol..

[bib41] Perisic O., Xiao H., Lis J.T. (1989). Stable binding of Drosophila heat shock factor to head-to-head and tail-to-tail repeats of a conserved 5 bp recognition unit. Cell.

[bib42] Pirkkala L., Nykanen P., Sistonen L. (2001). Roles of the heat shock transcription factors in regulation of the heat shock response and beyond. FASEB J..

[bib43] Privalov P.L., Dragan A.I., Crane-Robinson C., Breslauer K.J., Remeta D.P., Minetti C.A. (2007). What drives proteins into the major or minor grooves of DNA?. J. Mol. Biol..

[bib44] Riva L., Koeva M., Yildirim F., Pirhaji L., Dinesh D., Mazor T., Duennwald M.L., Fraenkel E. (2012). Poly-glutamine expanded huntingtin dramatically alters the genome wide binding of HSF1. J. Huntingtons Dis..

[bib45] Rohs R., Jin X., West S.M., Joshi R., Honig B., Mann R.S. (2010). Origins of specificity in protein-DNA recognition. Annu. Rev. Biochem..

[bib46] Sandqvist A., Bjork J.K., Akerfelt M., Chitikova Z., Grichine A., Vourc'h C., Jolly C., Salminen T.A., Nymalm Y., Sistonen L. (2009). Heterotrimerization of heat-shock factors 1 and 2 provides a transcriptional switch in response to distinct stimuli. Mol. Biol. Cell.

[bib47] Scherz-Shouval R., Santagata S., Mendillo M.L., Sholl L.M., Ben-Aharon I., Beck A.H., Dias-Santagata D., Koeva M., Stemmer S.M., Whitesell L. (2014). The reprogramming of tumor stroma by HSF1 is a potent enabler of malignancy. Cell.

[bib48] Schrodinger, L.L.C. (2010). The PyMOL Molecular Graphics System, Version 1.3r1.

[bib49] Sorger P.K., Nelson H.C. (1989). Trimerization of a yeast transcriptional activator via a coiled-coil motif. Cell.

[bib50] Tammsalu T., Matic I., Jaffray E.G., Ibrahim A.F.M., Tatham M.H., Hay R.T. (2014). Proteome-wide identification of SUMO2 modification sites. Sci. Signal..

[bib51] Tateishi Y., Ariyoshi M., Igarashi R., Hara H., Mizuguchi K., Seto A., Nakai A., Kokubo T., Tochio H., Shirakawa M. (2009). Molecular basis for SUMOylation-dependent regulation of DNA binding activity of heat shock factor 2. J. Biol. Chem..

[bib52] Vihervaara A., Sergelius C., Vasara J., Blom M.A., Elsing A.N., Roos-Mattjus P., Sistonen L. (2013). Transcriptional response to stress in the dynamic chromatin environment of cycling and mitotic cells. Proc. Natl. Acad. Sci. U S A.

[bib53] Vihervaara A., Sistonen L. (2014). HSF1 at a glance. J. Cell Sci..

[bib54] Vuister G.W., Kim S.J., Orosz A., Marquardt J., Wu C., Bax A. (1994). Solution structure of the DNA-binding domain of Drosophila heat shock transcription factor. Nat. Struct. Biol..

[bib55] Wang Q.S., Zhang K.H., Cui Y., Wang Z.J., Pan Q.Y., Liu K., Sun B., Zhou H., Li M.J., Xu Q. (2018). Upgrade of macromolecular crystallography beamline BL17U1 at SSRF. Nucl. Sci. Tech..

[bib56] Westerheide S.D., Anckar J., Stevens S.M., Sistonen L., Morimoto R.I. (2009). Stress-inducible regulation of heat shock factor 1 by the deacetylase SIRT1. Science.

[bib57] Westerheide S.D., Raynes R., Powell C., Xue B., Uversky V.N. (2012). HSF transcription factor family, heat shock response, and protein intrinsic disorder. Curr. Protein Pept. Sci..

[bib58] Winn M.D., Ballard C.C., Cowtan K.D., Dodson E.J., Emsley P., Evans P.R., Keegan R.M., Krissinel E.B., Leslie A.G., McCoy A. (2011). Overview of the CCP4 suite and current developments. Acta Crystallogr. D Biol. Crystallogr..

[bib59] Wu C. (1995). Heat shock transcription factors: structure and regulation. Annu. Rev. Cell Dev. Biol..

[bib60] Xiao Z., Guo L., Zhang Y., Cui L., Dai Y., Lan Z., Zhang Q., Wang S., Liu W. (2020). Structural analysis of missense mutations occurring in the DNA-binding domain of HSF4 associated with congenital cataracts. J. Struct. Biol. X.

[bib61] Yamamoto N., Takemori Y., Sakurai M., Sugiyama K., Sakurai H. (2009). Differential recognition of heat shock elements by members of the heat shock transcription factor family. FEBS J..

