# Supplementary material for: Structures of heat shock factor trimers bound to DNA
Source: iScience. 2021 Aug 5;24(9):102951. doi: 10.1016/j.isci.2021.102951 (PMC8379338; doi:10.1016/j.isci.2021.102951)
Supplement: Document S1. Figures S1–S7 and Tables S1–S4 [file mmc1.pdf]

iScience, Volume *24*

Supplemental information

Structures of heat shock factor trimers bound to DNA

Na Feng, Han Feng, Sheng Wang, Avinash S. Punekar, Rudolf Ladenstein, Da-Cheng Wang, Qinghua Zhang, Jingjin Ding, and Wei Liu

Supplemental Data Items

Table S1 Nucleotide sequences used in crystallization and structure determination, Related to STAR Methods

**Protein-DNA complexes**	**Nucleotide sequences***
HSF2 with 2-site HSE (12 bp)	5’-ggGAAtaTTCcc-3’<br>3’-ccCTTatAAGgg-5’
HSF1 with 2-site HSE (14 bp)	5’-gccGAAtaTTCggc-3’<br>3’-cggCTTatAAGccg-5’
HSF2 with 3-site HSE (20 bp + 1-nt overhang)	5’-*t*gcgTTCtaGAAtaTTCgcgg -3’<br>3’- cgcAAGatCTTatAAGcgcc*a*-5’
HSF1 with 3-site HSE (22 bp + 1-nt overhang)	5’-*t*ggcgTTCtaGAAtaTTCgcgga -3’<br>3’- ccgcAAGatCTTatAAGcgcct*a*-5’
HSF1 with 3-site HSE (23 bp + 1-nt overhang)	5’-tgtgcgTTCtaGAAtaTTCgcgag -3’<br>3’- cacgcAAGatCTTatAAGcgctca-5’

* Uppercased are the conserved triplets (GAA or TTC) responsible for sequence-specific binding with HSFs, and italic are the nucleotides used as 5’-overhangs.

Table S2 Optimal conditions for crystal growth and beamlines for data collection at SSRF, Related to STAR Methods

**Cocrystals**	**Optimal conditions**	**Beamlines***
HSF2-DBD bound to 2-site HSE (head-to-head)	0.2 M lithium citrate and 23% (w/v) PEG 3350	BL17U1 (0.97915 Å)
HSF1-DBD bound to 2-site HSE (head-to-head)	0.1 M Tris-HCl, pH 7.8 and 28% (w/v) PEG 3350	BL18U1 (1.00000 Å)
HSF2-DBD bound to 3-site HSE	0.1 M HEPES pH 7.5 and 25% (w/v) PEG 3350	BL18U1 (0.97736 Å)
HSF1-DBD bound to 3-site HSE (22 bp + 1-nt overhang)	2% v/v Tacsimate pH 6.0，0.1 M Bis-Tris pH 6.4 and 21% (w/v) PEG 3350	BL18U1 (0.97778 Å)
HSF1-DBD bound to 3-site HSE (23 bp + 1-nt overhang)	2% v/v Tacsimate pH 6.0，0.1 M Bis-Tris pH 6.5 and 21% (w/v) PEG 3350	BL17U1 (0.97949 Å)

* The values in parenthesis are the wavelengths used for data collection.

Table S3 RMSDs among the copies[a] in HSF1 trimer-binding structures #1 (containing a 23 bp DNA duplex) and #2 (containing a 24 bp DNA duplex), Related to Figure 3

	**Copy #2 in structure #1**	**Copy #1 in structure #2**	**Copy #2 in structure #2**
**Protein-protein RMSDs[b]**			
Copy #1 in structure 1 (Å)	0.25 Å	0.39 Å	0.38 Å
Copy #2 in structure 1 (Å)		0.37 Å	0.33 Å
Copy #1 in structure 2 (Å)			0.30 Å
**DNA-DNA RMSDs[c]**			
Copy #1 in structure 1 (Å)	0.18 Å	0.18 Å	0.24 Å
Copy #2 in structure 1 (Å)		0.27 Å	0.20 Å
Copy #1 in structure 2 (Å)			0.31 Å

[a] One copy includes three DBDs and a DNA duplex containing three HSE repeats.

[b] RMSDs are calculated on all atoms including main chain and side chain atoms in the DBDs.

[c] RMSDs are calculated on all atoms including phosphate backbone, ribose and base atoms in the DNA molecules.

Table S4 Protein-protein interactions among HSF-DBDs revealed in the co-crystals of HSF1/2-DBDs bound to 3-site HSE[*], Related to Figure 5

**Complex**	**DBD pair**	**Interface residues**	**Interface area (Å²)**	**Hydrogen bonds**	**Van der Waals contacts**
HSF2-DBDs bound to 3-site HSE (20 bp + 1-nt overhang)	*I - II*	10 : 11	239:241	1	22
	II - III	3 : 2	86 : 88	2	9
	I - III	7 : 12	351 : 287	6	35
HSF1-DBDs bound to 3-site HSE (22 bp + 1-nt overhang) (HSE-*I*)	*I - II*	6 : 6	78 : 72	0	0
	II - III	2 : 2	83 : 77	2	8
	I - III	9 : 12	352 : 281	6	30
HSF1-DBDs bound to 3-site HSE (22 bp + 1-nt overhang) (HSE-*II*)	*I' – II'*	10 : 10	160 : 142	1	4
	II' – III'	2 : 2	82 : 73	2	8
	I' – III'	10 : 11	294 : 261	5	39
HSF1-DBDs bound to 3-site HSE (23 bp + 1-nt overhang) (HSE-*I*)	*I - II*	9 : 9	130 : 126	1	1
	II - III	2 : 2	87 : 79	2	9
	I - III	8 : 12	342 : 287	5	34
HSF1-DBDs bound to 3-site HSE (23 bp + 1-nt overhang) (HSE-*II*)	*I' – II'*	9 : 10	150 : 135	1	2
	II' – III'	3 : 3	89 : 76	2	9
	I' – III'	10 : 11	356 : 286	4	40

[*] All values given in this table were calculated using the *PDBePISA* server at European Bioinformatics Institute(Krissinel and Henrick, 2007).

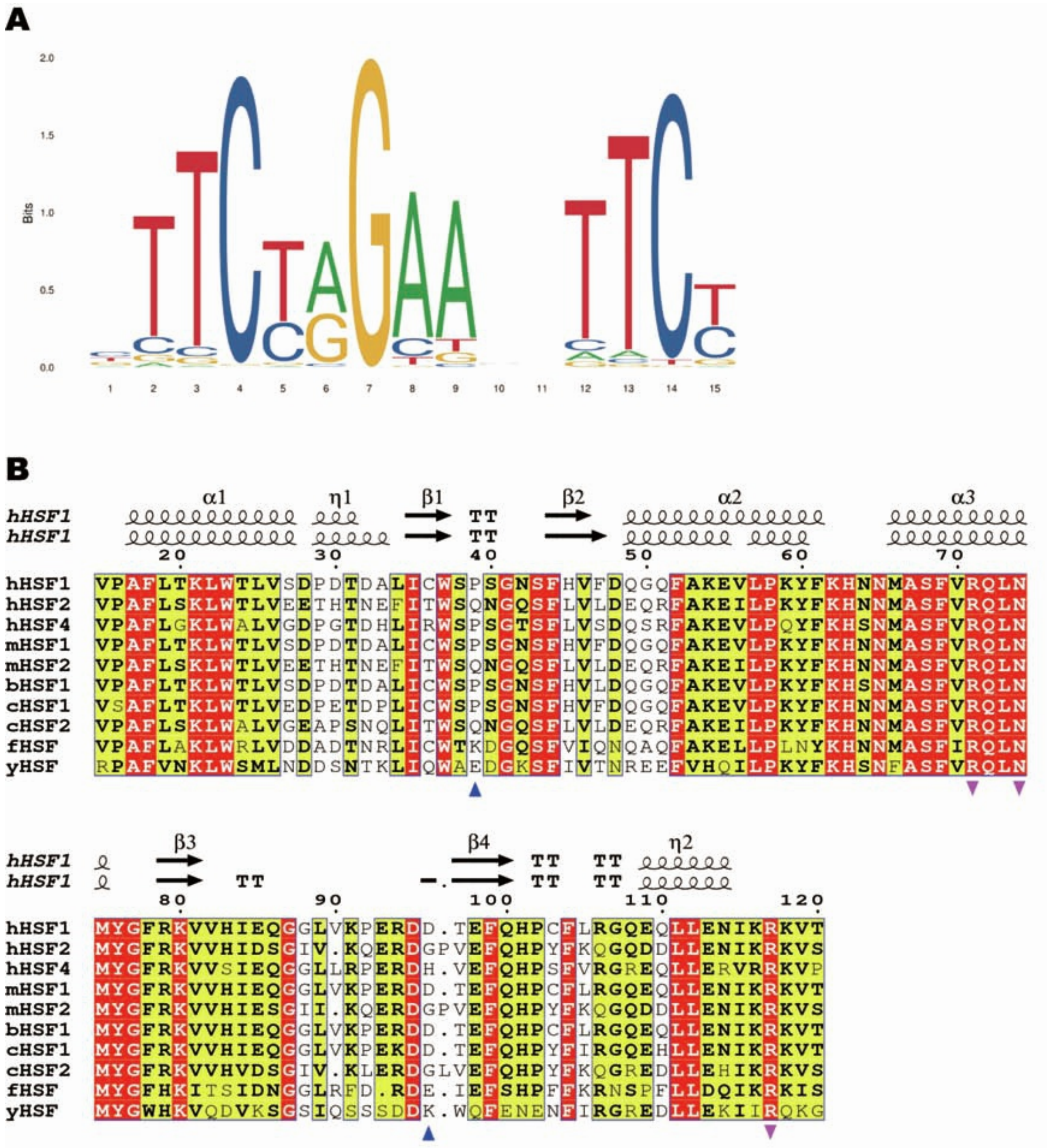

Figure S1 The consensus HSE sequence recognized by HSFs and amino acid sequence alignment of the DBD, Related to Introduction and Table 1. (**A**), The sequence logo of the canonical HSE bound by HSF1, according to the Chip-seq data in the latest version of JASPAR database(Khan et al., 2018). (**B**), Amino acid sequence alignment of the DBDs among human HSF1 (hHSF1), HSF2 (hHSF2), HSF4 (hHSF4), murine HSF1 (mHSF1), HSF2 (mHSF2), bovine HSF1 (bHSF1), chicken HSF1 (cHSF1), HSF2 (cHSF2), fruit fly HSF (fHSF) and yeast HSF (yHSF). The highly conserved amino acids, including Arg71, Asn74, Arg117 in hHSF1 and Arg63, Asn66, Arg109 in hHSF2, that make the sequence-specific contacts with nucleobases in the major groove are highlighted by purple downward triangles. Pro39 and Asp96 in HSF1 that may render the wing domain less flexible are labeled with blue upward triangles. On the top of sequence alignment, the secondary structures of DNA-free and DNA-bound hHSF1 are schematically shown in the first and second lines, respectively.

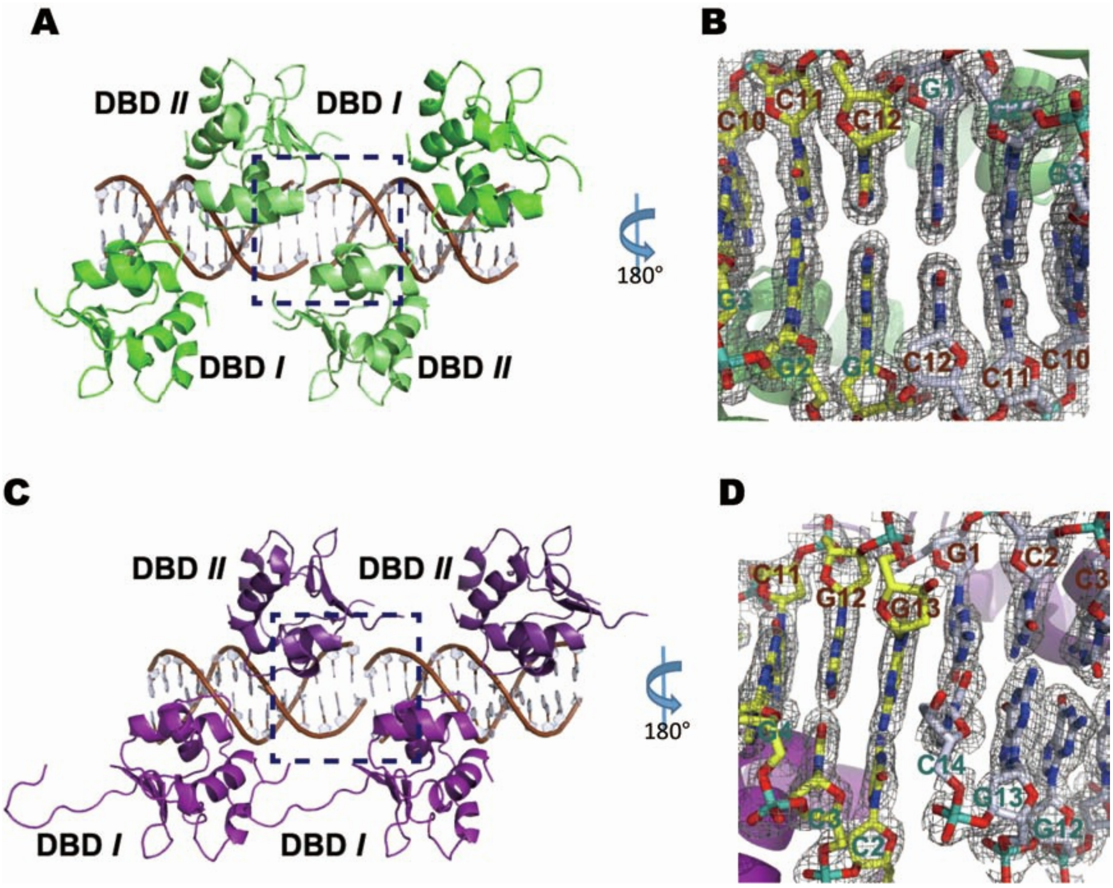

Figure S2 Packing of macromolecules in the complex crystal of HSF1/2-DBD with a two-site head-to-head HSE, Related to Figure 1. (**A**), Two blunt-ended HSEs (12 bp) bound by HSF2-DBD from neighboring asymmetric units are packed in a tail-to-tail manner. (**B**), Close view of the region encircled by a dashed box in (**A**). (**C**), Two blunt-ended HSEs (14 bp) bound by HSF1-DBD from neighboring asymmetric units are imperfectly in a head-to-tail manner with unpaired terminal nucleotides. (**D**), Close view of the region encircled by a dashed box in (**C**). In panels (**B**) and (**D**), The backbones of two symmetrically related HSEs are colored in pale blue and yellow. Nucleotides in different DNA strands are labeled in brown and cyan color. The $2F_O - F_C$ electron density shown in (**B**) and (**D**) was contoured at 1.0 σ.

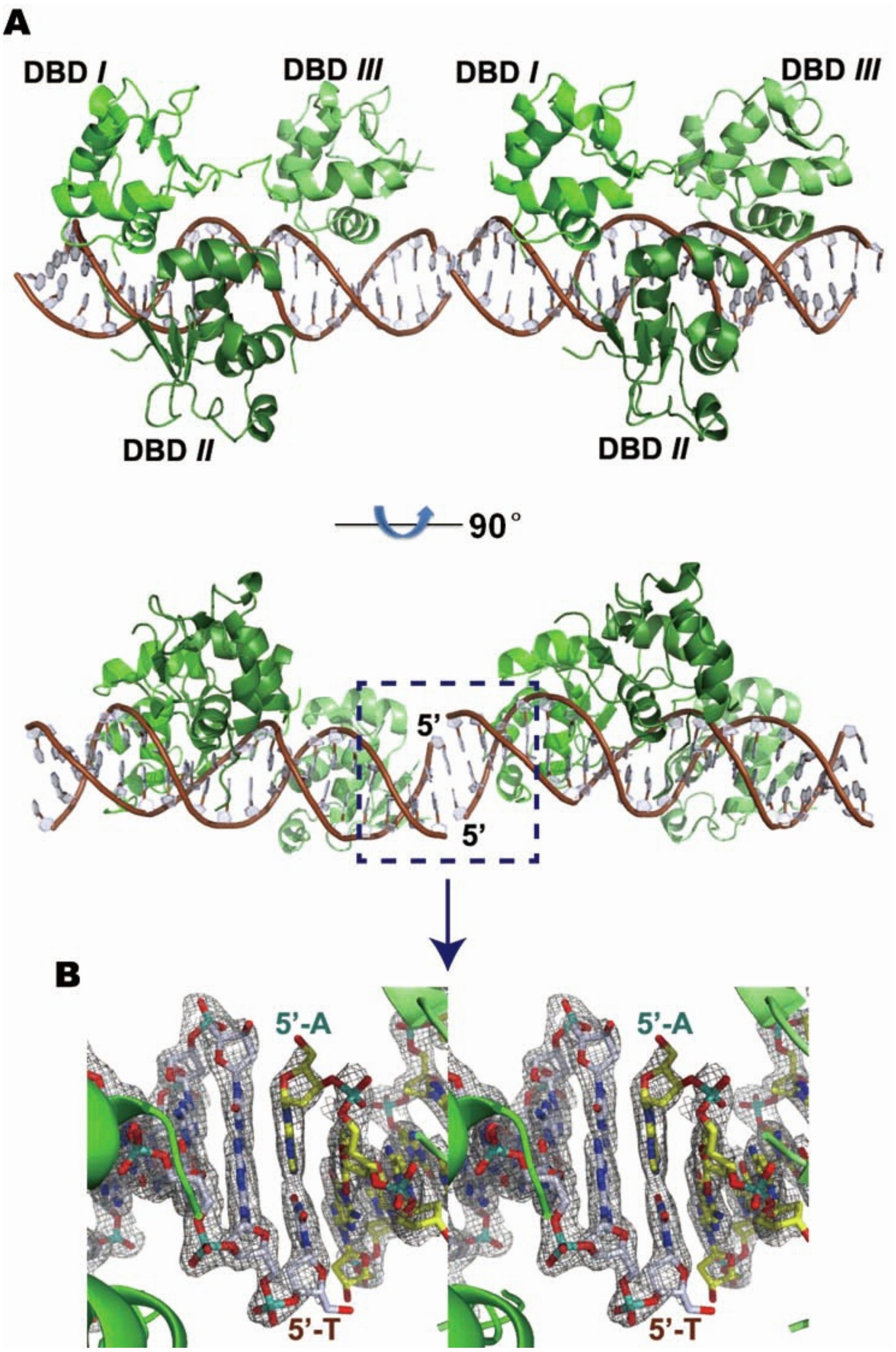

Figure S3 Packing of macromolecules in the complex crystal of HSF2-DBD with a 3-site HSE, Related to Figure 2. (**A**), Two sticky-ended HSEs (20 bp with a 5'-overhang) present in neighboring asymmetric units are arranged like an intact DNA duplex in the crystal. (**B**), Close view of the region encircled by a dashed box in (**A**). The backbones of two symmetrically related HSEs are colored in pale blue and yellow. Nucleotides in different DNA strands are labeled in brown and cyan color. The $2F_O - F_C$ electron density shown in (**B**) was contoured at 1.0 σ.

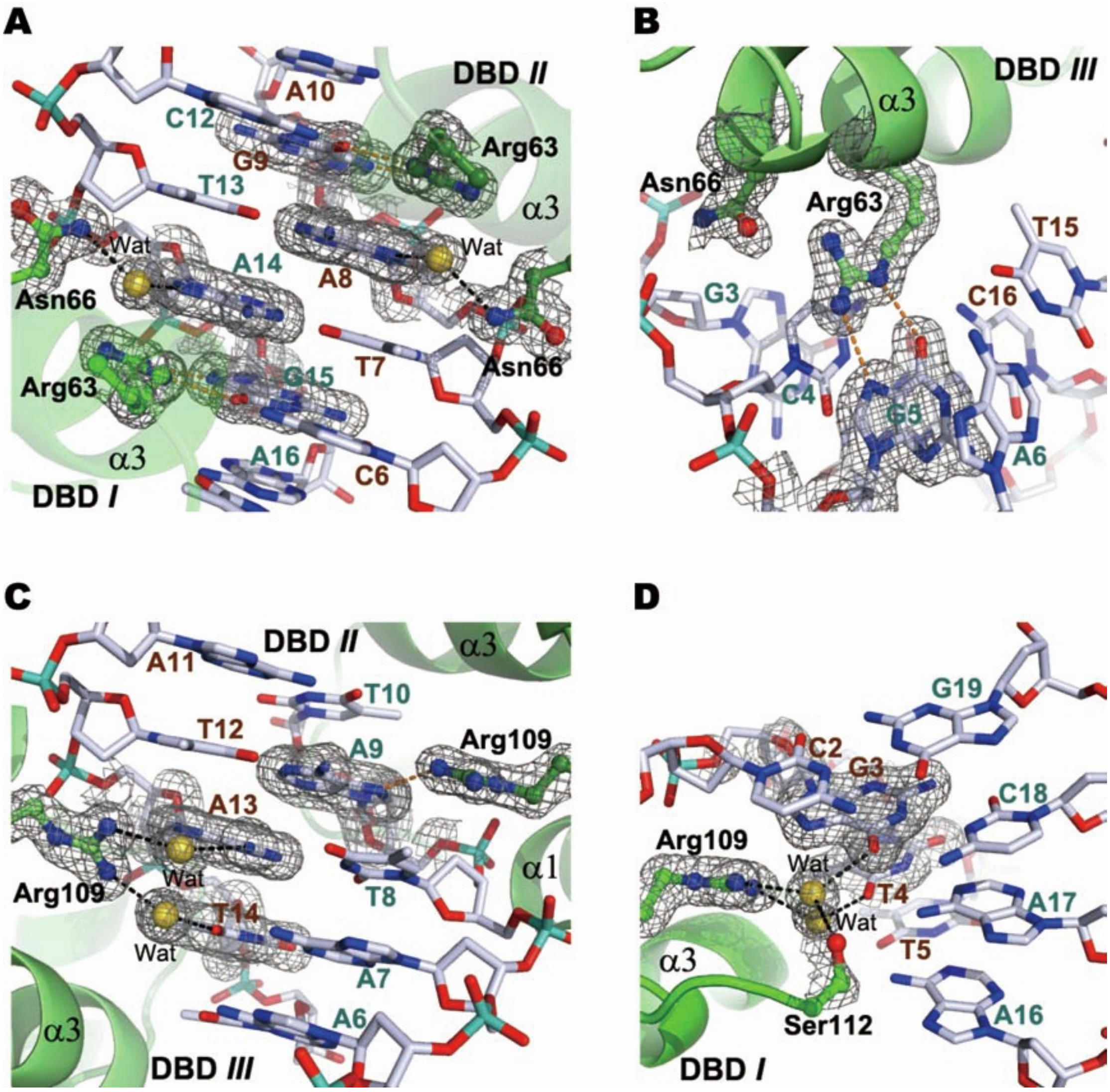

Figure S4 Detailed protein-DNA interactions present in the complex crystal of of HSF2-DBD bound to a three-site HSE, Related to Figure 2. (**A**, **C** and **D**). Same representation of Figure 2d, 2e and 2f, but shown with the 2F_o-F_c electron density contoured at 1.0 σ. (**B**), Interactions occurring in the major groove between nucleotides and helix α3 of DBD-*III*.

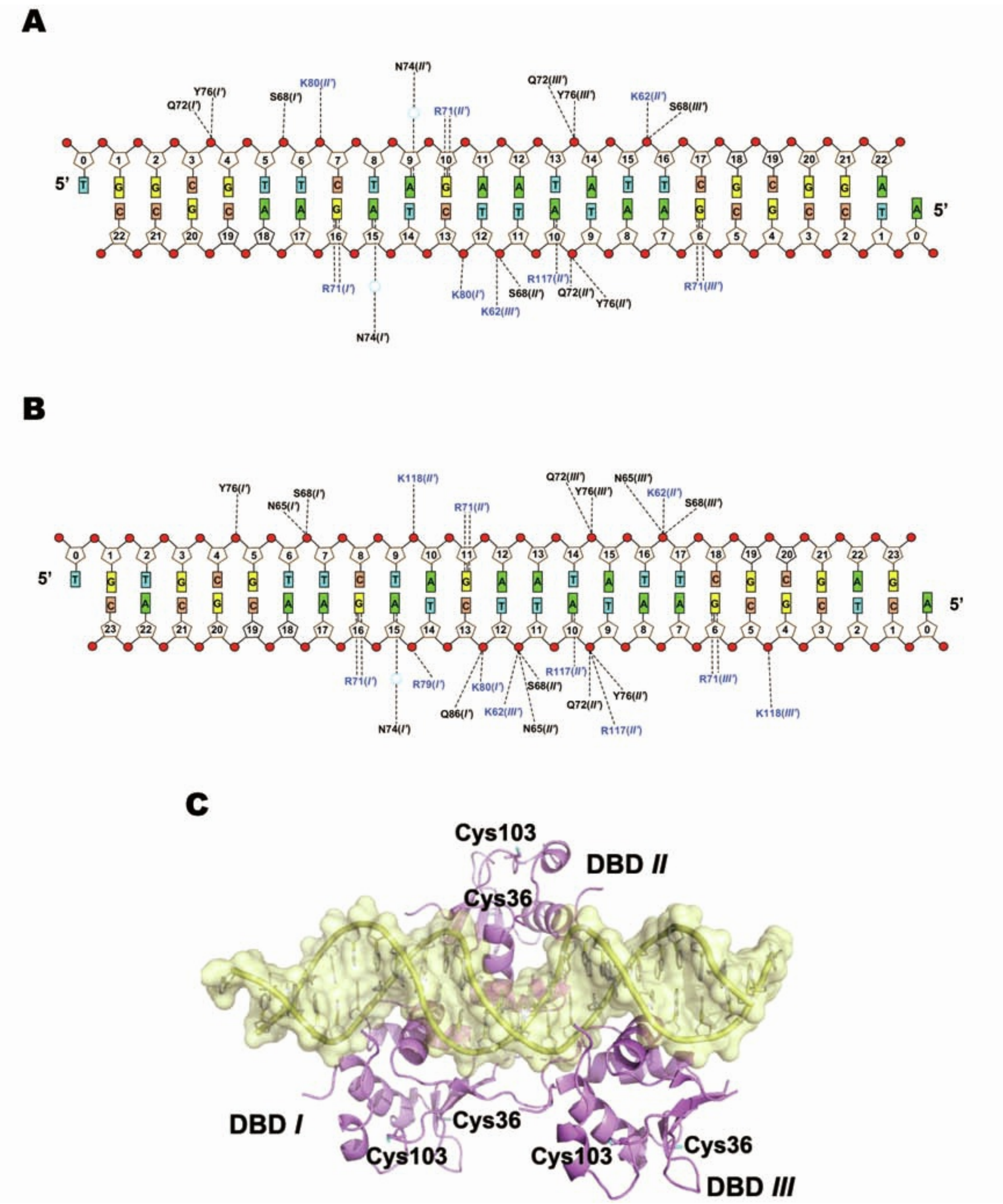

Figure S5 Structures of HSF1-DBD bound to DNA duplexes comprising 3 binding sites, Related to Figure 3. (**A** and **B**), Schematic drawings of the protein-DNA interactions occurring around HSE-*II* in cocrystals containing 22 bp DNA with a 5'-overhang (**A**) or 23 bp DNA with a 5'-overhang (**B**), as supplementary drawings for Figure 3C-D. (**C**) The locations of two cysteine residues, Cys36 and Cys103 in HSF1-DBDs, which rules out the possibility of intramolecular or intermolecular disulfide bond formation.

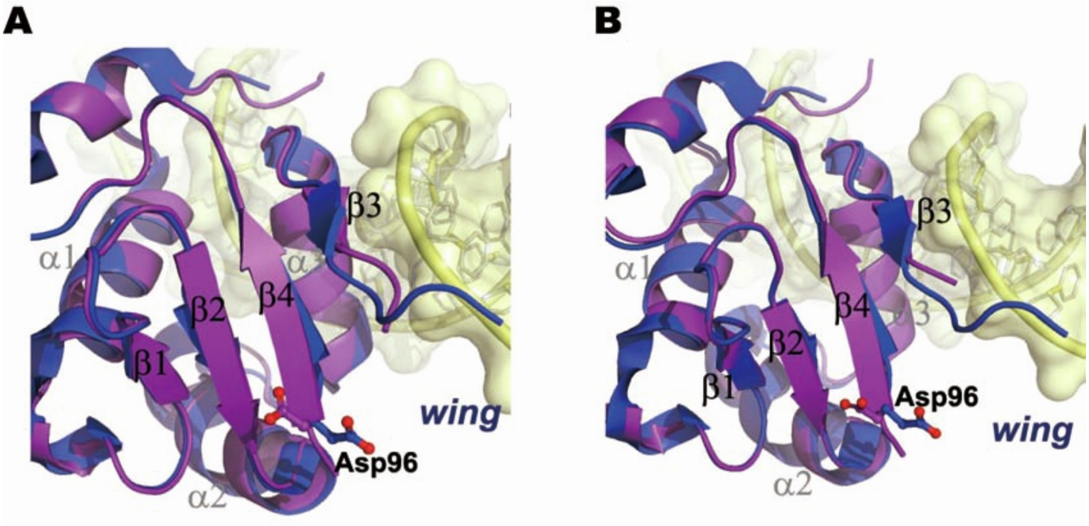

Figure S6 Comparison of HSF1-DBD from different crystal structures, Related to Figure 6. (**A** and **B**), Superimposition of the DNA-free structure (PDB ID 5HDG) and DBD *II* (**A**) or DBD *III* (**B**) in the trimer-binding structure comprising a 23 bp DNA duplex. The DNA-complexed and DNA-free structures are colored in purple and blue, respectively.

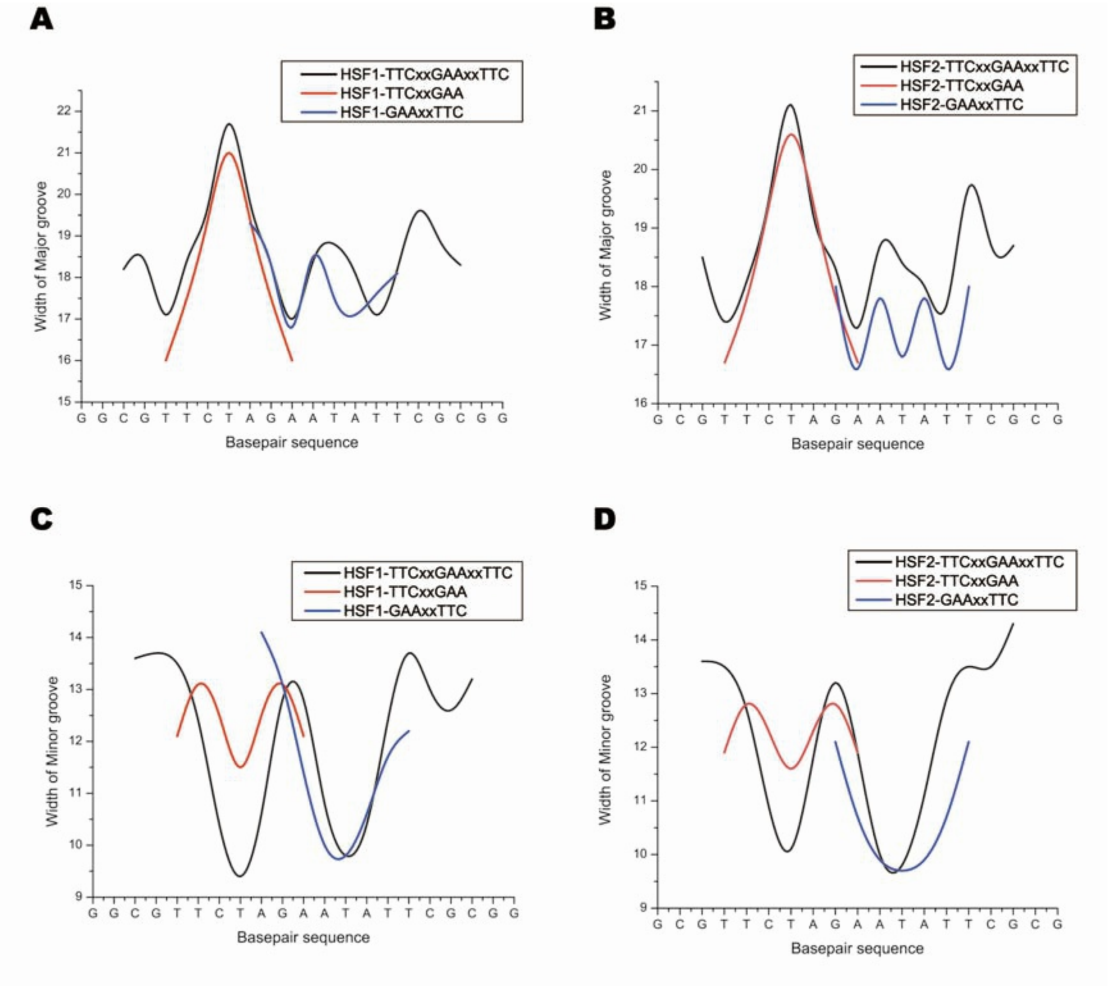

Figure S7 Comparisons of the major and minor groove widths of the DNA molecules present in different crystals, Related to Figure 7. (**A** and **B**), The major groove widths of the DNA duplexes containing 2-site HSEs arranged in the tail-to-tail (red) and head-to-head (blue) orientations, and 3-site HSEs (black) bound by HSF1 (**A**) or HSF2 (**B**). (**C** and **D**), The minor groove widths of the DNA duplexes containing 2-site HSEs arranged in the tail-to-tail (red) and head-to-head (blue) orientations, and 3-site HSEs (black) bound by HSF1 (**C**) or HSF2 (**D**). In (**A** and **B**), the structure containing the 23 bp DNA was used in this analysis.
